# Tularemia treatment: experimental and clinical data

**DOI:** 10.3389/fmicb.2023.1348323

**Published:** 2024-01-17

**Authors:** Max Maurin, Léa Pondérand, Aurélie Hennebique, Isabelle Pelloux, Sandrine Boisset, Yvan Caspar

**Affiliations:** ^1^Centre National de Référence Francisella tularensis, CHU Grenoble Alpes, Grenoble, France; ^2^Université Grenoble Alpes, Translational Innovation in Medicine and Complexity (TIMC), Centre National de la Recherche Scientifique (CNRS), Grenoble, France; ^3^Université Grenoble Alpes, Commissariat à l’énergie atomique (CEA), CNRS, Institut de Biologie Structurale (IBS), Grenoble, France

**Keywords:** tularemia, *Francisella tularensis*, treatment, antibiotic susceptibility, animal models, human infections

## Abstract

Tularemia is a zoonosis caused by the Gram negative, facultative intracellular bacterium *Francisella tularensis*. This disease has multiple clinical presentations according to the route of infection, the virulence of the infecting bacterial strain, and the underlying medical condition of infected persons. Systemic infections (e.g., pneumonic and typhoidal form) and complications are rare but may be life threatening. Most people suffer from local infection (e.g., skin ulcer, conjunctivitis, or pharyngitis) with regional lymphadenopathy, which evolve to suppuration in about 30% of patients and a chronic course of infection. Current treatment recommendations have been established to manage acute infections in the context of a biological threat and do not consider the great variability of clinical situations. This review summarizes literature data on antibiotic efficacy against *F. tularensis in vitro*, in animal models, and in humans. Empirical treatment with beta-lactams, most macrolides, or anti-tuberculosis agents is usually ineffective. The aminoglycosides gentamicin and streptomycin remain the gold standard for severe infections, and the fluoroquinolones and doxycycline for infections of mild severity, although current data indicate the former are usually more effective. However, the antibiotic treatments reported in the literature are highly variable in their composition and duration depending on the clinical manifestations, the age and health status of the patient, the presence of complications, and the evolution of the disease. Many patients received several antibiotics in combination or successively. Whatever the antibiotic treatment administered, variable but high rates of treatment failures and relapses are still observed, especially in patients treated more then 2–3 weeks after disease onset. In these patients, surgical treatment is often necessary for cure, including drainage or removal of suppurative lymph nodes or other infectious foci. It is currently difficult to establish therapeutic recommendations, particularly due to lack of comparative randomized studies. However, we have attempted to summarize current knowledge through proposals for improving tularemia treatment which will have to be discussed by a group of experts. A major factor in improving the prognosis of patients with tularemia is the early administration of appropriate treatment, which requires better medical knowledge and diagnostic strategy of this disease.

## 1 Introduction

*Francisella tularensis* is a Gram-negative, facultative intracellular bacterium. Only two subspecies cause tularemia: subsp. *tularensis* (type A) in Northern America, and subsp. *holarctica* (type B) in the whole Northern hemisphere and southern Australia (Sjöstedt, 2007; Jackson et al., 2012). *F. tularensis* subsp. *mediasiatica* is restricted to specific areas in Central Europe and Russia and has never been isolated from humans (Ellis et al., 2002; Timofeev et al., 2017). *F. tularensis* is highly virulent and belongs to the category A of potential bioterrorism agents of the Centers for Diseases Control and Prevention (CDC, USA) [CDC | Bioterrorism Agents/Diseases (by category) | Emergency Preparedness & Response, 2019]. Type A and type B strains are split into clades and subclades, the major clades being A1, A2, B4, B6, B12, and B16 (Johansson et al., 2004; Vogler et al., 2009).

*Francisella tularensis* has been detected in more than 200 animal species, including most mammals and arthropod vectors (*Ixodidae* ticks, and mosquitoes in Sweden and Finland) (Telford and Goethert, 2020). This bacterium can also survive for prolonged periods in the hydrotelluric environment (Hennebique et al., 2019). Human infection with *F. tularensis* can occur through the skin (e.g., skin wound after contact with an infected animal, and arthropod bites), the conjunctiva, the oral route (e.g., ingestion of contaminated food or water), or the respiratory route (inhalation of a contaminated aerosol). After a short incubation period (usually 3–5 days), infected persons usually develop a flu-like illness (Tärnvik and Chu, 2007; Hepburn and Simpson, 2008; Maurin and Gyuranecz, 2016). Then, the disease may evolve to six classical forms: the ulceroglandular (UG) and glandular (GL) forms, a regional lymphadenopathy, with or without detectable skin inoculation lesion, respectively; the oculoglandular (OG) form, a conjunctivitis with satellite lymphadenopathy; the oropharyngeal (OP) form, a pharyngitis with regional lymphadenopathy; the pneumonic (PN) form, an acute, subacute, or chronic pneumonia; and the typhoidal (TY) form, mimicking typhoid. Many complications may occur (e.g., soft tissue infection, meningitis, endocarditis, and prosthetic joint infection), sometimes as inaugural presentations of the disease (Tärnvik and Chu, 2007; Hepburn and Simpson, 2008; Maurin and Gyuranecz, 2016). The global death rate of tularemia is less than 1% for type B infections and about 2–3% for type A, but can be up to 30% for untreated acute type A pneumonia (Dennis et al., 2001).

Human tularemia mostly occur as sporadic cases but outbreaks caused by one or several *F. tularensis* clones are regularly reported (Pérez-Castrillón et al., 2001; Reintjes et al., 2002; Celebi et al., 2006; Kantardjiev et al., 2006; Svensson et al., 2009; Hauri et al., 2010; Larssen et al., 2011; Johansson et al., 2014). The number of reported tularemia cases has increased in recent years in most endemic countries. The European Center for Disease Control and Prevention (ECDC) reported 641 confirmed human tularemia cases in Europe in 2020 (incidence, 0.15 per 100,000 persons per year), most of which occurred in Sweden and Finland. In the USA, 1984 human tularemia cases were reported from 2011 to 2019 (average incidence, 0.07 cases per 100,000 person per year), predominantly in Arkansas, Missouri, Oklahoma, and Kansas (Bishop et al., 2023). Tularemia is a significant public health problem since the 2000’s in Turkey (Erdem et al., 2014).

Tularemia diagnosis can be established serologically or by culture or PCR detection of *F. tularensis* from clinical samples (Tärnvik and Chu, 2007; Hepburn and Simpson, 2008; Maurin, 2020; Wawszczak et al., 2022). Culture and PCR tests may allow an early and specific diagnosis of tularemia. Depending on the clinical presentation, *F. tularensis* can be grown or PCR-detected from various clinical samples including blood, skin eschar, conjunctival or pharyngeal exudates, lymph node samples, cerebrospinal fluid, sputum, and osteoarticular samples. However, the sensitivity of *F. tularensis* culture rapidly decreases over time and this bacterium is isolated in only about 10% of tularemia cases. PCR tests usually remain positive for longer time. For example, they are usually positive for lymph node samples collected after several weeks evolution of tularemia while culture is usually negative. However, in many patients, clinical samples other than blood and serum samples are not available for PCR testing. Therefore, tularemia diagnosis remains often based on serology. Significant titers of specific antibodies are usually detected only 2–3 weeks after disease onset. False positive results may arise from long term persistence (months to years) of antibodies in persons with past tularemia infection and from serological cross reactions. Therefore, two serum samples taken at least 2 weeks apart should be tested to increase specificity. Tularemia diagnosis is usually considered confirmed in patients with compatible epidemiological and clinical data and *F. tularensis* detection by culture or PCR, or a seroconversion or a 4-fold increase of antibody titers at 2 weeks or more interval. In the same context, a single positive serum sample correspond to a probable tularemia case.

Tularemia diagnostic and treatment delay is the rule in most endemic countries, due to poor specificity of clinical symptoms and late medical consultation of patients with mild diseases. No vaccine against tularemia is authorized in humans or animals. Prophylaxis is based on protective measures against sources of infection (e.g., animals, arthropods, drinking water and food, hydrotelluric environment). Appropriate recommendations should be provided by physicians to people living in tularemia endemic areas and at high risk of *F. tularensis* infections due to their occupation (e.g., farmers, veterinarians, butchers, landscapers) or leisure activities (e.g., hunting, trapping, walking). Only aminoglycosides, tetracyclines and fluoroquinolones are considered in the first-line treatment of tularemia. However, the current international recommendations for tularemia treatment have been primarily developed to deal with emergency situations in the context of bioterrorism (Dennis et al., 2001; Bossi et al., 2004). This review updates data we previously published on antibiotic susceptibilities of *F. tularensis* (Caspar and Maurin, 2017) and summarizes the current literature on efficacy of antibiotics in *F. tularensis*-infected animal models and tularemia patients. Our objective is to highlight the variability and complexity of clinical situations encountered in patients with tularemia, which should lead to new therapeutic recommendations to reduce the disabling and chronic nature of this disease, as well as the risk of severe complications. Although this study does not allow us to propose new therapeutic recommendations, we have attempted to provide suggestions for improving the treatment of tularemia based on the medical context.

## 2 Search strategy and selection criteria

Data were collected from English literature in Pubmed database using the following keywords: *Francisella tularensis* or tularemia, and one of the following terms, antibiotics, treatment, antibiotic susceptibility, antibiotic resistance, cell models, animal models, pneumonia, endocarditis, aortitis, meningitis, encephalitis, ocular infections, osteoarthritis, soft tissue infections, skin rash, skin infections, children, pregnant woman, and immunocompromised. All article types dealing with antibiotic susceptibility of *F. tularensis*, or tularemia treatment were selected. Some tularemia reviews were also included in this bibliography. In total, 222 articles were selected for this review.

## 3 Antibiotic activity against *F. tularensis in vitro*

Data on antibiotic susceptibility of *F. tularensis* to antibiotics have been previously reviewed by Caspar and Maurin (2017). Therefore, the present review will only summarize the most significant data, adding those published in more recent years.

### 3.1 Antibiotic activity in a cellular media

#### 3.1.1 Methods

*Francisella tularensis* antibiotic susceptibility has been mainly evaluated using the broth microdilution (BMD) and the E-test strips (E-test) methods, and less frequently the antibiotic agar dilution method (Caspar and Maurin, 2017). The BMD method from the Clinical and Laboratory Standards Institute (CLSI) is currently the best standardized method (Clinical And Laboratory Standards Institute, 2005, 2016). Experimental conditions include the use of cation-adjusted Mueller Hinton broth (caMHB) supplemented with 2% defined growth supplement (e.g., Polyvitex^®^ or IsoVitalex^®^), a 5 × 10^5^ cfu/ml bacterial inoculum, and a 48 h incubation of cultures at 37°C in aerobic atmosphere (or 5% CO_2_ atmosphere for capnophilic strains). In Tables 1–4 we have summarized data obtained with the BMD method using CaMHB medium or the E-test strips method. Some publications reporting high MICs suggesting acquired resistance to antibiotics in *F. tularensis* but without genetic confirmation were not considered (Baker et al., 1985; Scheel et al., 1993; Martínez-Martínez et al., 2021).

**TABLE 1:** MICs of beta-lactams against *Francisella tularensis.*

References	*F. tularensis* strains			Method / medium	Beta-lactams MICs (mg/L)									
	Country source	Type (number)	Isolated in		PnG	PnA	AMC	PTZ	CRO	CAZ	FEP	IMP	MPN	AZT
Georgi et al., 2012	Europe	B (69)	NA	BMD / caMHB + 2% IsoVx	>0.5		>64			>32		>16		
Heine et al., 2017	Variable	A (19), B (10)	1920–2002	BMD / caMHB + 2% IsoVx	2− > 64	4− > 64	0.5− > 64		0.03− > 64*		0.12− > 64	0.06− > 64	0.12− > 64	0.5− > 64
Caspar et al., 2018	France	B6 (59)	2006–2017	BMD / caMHB + 2% IsoVx		64- ≥ 128							>32	
All BMD tests					2− > 64	4- ≥ 128	0.5− > 64		0.03− > 64	>32	0.12− > 64	0.06− > 64	0.12− > 64	0.5− > 64
Hotta et al., 2013	Japan	B (36)	1926–1989	E-test/ Chocolate II	64− > 256				0.047− > 256*			0.047− > 256	0.094− > 256	0.75− > 256
Yesilyurt et al., 2011	Turkey	B bvII (39)	2009–2010	E-test / GCBA		>256	>256	>256	>256	>256	>256	>32		
Tomaso et al., 2005	Austria	B bvII (50)	1992–1998	E-test / CHAB		>256		>256	>32	>256	>32	0.5− > 32	1.5− > 32	>256
Ikäheimo et al., 2000	Finland	B (38)	NA	E-test / CHAHb				>256	>32	>256		>32	>32	
Velinov et al., 2011	Bulgaria	B bvII (21)	NA	E-test / caMHB + 2% IsoVx	>256			>256	2–4					
All E-tests					64− > 256	> 256	>256	>256	0.047− > 256	>256	>32− > 256	0.047− > 256	0.094− > 256	0.75− > 256

Antibiotics: penicillin G (PnG), ampicillin or amoxicillin (PnA), amoxicillin/clavulanate (AMC, ratio 2:1), piperacillin/tazobactam (PTZ, ratio 8:1 usually), ceftriaxone (CRO), ceftazidime (CAZ), cefepime (FEP), imipenem (IMP), meropenem (MPN), aztreonam (AZT). Methods: BMD: broth microdilution technique; E-test: E-test strip method. Culture media: caMHB: cation-adjusted MHB; GCBA: glucose/cysteine blood agar (i.e., brain heart infusion agar supplemented with 1% glucose monohydrate, 0.1% L-cysteine, and 9% chocolatized sheep blood); CHA: cysteine heart agar; CHAB: CHA + 10% sheep blood; CHAHb: CHA + 2% hemoglobin; IsoVx: IsoVitaleX™.

*The MIC ranges were the same for cefotaxime and ceftriaxone; NA: data not available

#### 3.1.2 Bacteriostatic activity (MIC and MIC90)

The beta-lactams are poorly active against *F. tularensis* with MIC90 (i.e., MIC inhibiting 90% of the tested strains) highest than the maximum concentration tested (i.e., > 32–256 mg/L) for penicillins, cephalosporins, monobactams, and carbapenems (Table 1). A few *F. tularensis* strains displayed low MICs, e.g., 4 mg/L for penicillin A, 0.03 mg/L for ceftriaxone, 0.047 mg/L for imipenem, and 0.094 mg/L for meropenem (Tomaso et al., 2005; Hotta et al., 2013; Heine et al., 2017). In the above studies, the percentage of strains with MICs lower than 2 mg/L among those tested varied from 4 % (Tomaso et al., 2005) up to 66.6% (Hotta et al., 2013) for imipenem, and was 50% for ceftriaxone (Hotta et al., 2013). These two studies used the E-test method. The last study did not specify the distribution of strains according to the MIC (Heine et al., 2017). These data are difficult to interpret until we further understand the mechanisms of beta-lactam resistance in *F. tularensis* (see below).

The beta-lactam/beta-lactamase inhibitor combinations are no more effective (e.g., amoxicillin/clavulanate and piperacillin/tazobactam) (Ikäheimo et al., 2000; Tomaso et al., 2005; Velinov et al., 2011; Yesilyurt et al., 2011; Georgi et al., 2012; Heine et al., 2017). Recently, ceftobiprole medocaril has been reported to display an MIC of < 0.03 mg/L for the Schu S4 strain (Hahn et al., 2023).

The aminoglycosides gentamicin and streptomycin are highly active against *F. tularensis in vitro* (Table 2). All tested strains had MICs lower than the CLSI breakpoints (≤8 mg/L for streptomycin and ≤ 4 mg/L for gentamicin). Tobramycin displayed MIC and MIC90 ranges of ≤ 0.03–0.5 mg/L and 0.25–2 mg/L for the BMD method and 0.125–3 mg/L and 0.25–1.5 mg/L for the E-test method (Ikäheimo et al., 2000; Tomaso et al., 2005; Yesilyurt et al., 2011; Heine et al., 2017; Caspar et al., 2018). Amikacin was less effective with MIC and MIC90 ranges of ≤ 0.03–8 mg/L and 2 mg/L for the MBD method and 0.75–16 mg/L and 1–4 mg/L for the E-tests method (Tomaso et al., 2005; Yesilyurt et al., 2011; Heine et al., 2017).

**TABLE 2:** MICs of aminoglycosides against *Francisella tularensis.*

References	*F. tularensis* strains			Method/medium	Gentamicin MICs (mg/L)		Streptomycin MICs (mg/L)	
	Country source	Type (number)	Isolated in		Range	MIC90	Range	MIC90
Origgi et al., 2014	Switzerland	B6 (19)	1996–2013	BMD / caMHB + 2% IsoVx	≤0.12–0.25	0.25	4	4
		B12 (5)			≤0.12–0.25		4	
Georgi et al., 2012	Europe	B (69)	NA	BMD / caMHB + 2% IsoVx	≤0.25–0.5	0.5	≤0.5–2	
	North America	A (7)			≤0.25–0.5		≤2	
Urich and Petersen, 2008	North America	A (92)	1974–2005	BMD / caMHB + 2% IsoVx	0.03–0.5	0.25	0.25–4	2
		B (77)			0.03–0.5	0.12	0.25–2	2
Heine et al., 2017	Variable	A (19), B (10)	1920–2002	BMD / caMHB + 2% IsoVx	≤0.03–1	0.5		
Caspar et al., 2018	France	B6 (59)	2006–2017	BMD / caMHB + 2% IsoVx	≤0.03–0.25	0.125		
All BMD tests					≤0.03–1	0.12–0.5	0.25–4	2–4
Johansson et al., 2000	Sweden	B (7)	1998	E-test / mThayer-Martin	0.5–1			
Hotta et al., 2013	Japan	B (36)	1926–1989	E-test/ Chocolate II agar	0.023–0.5	0.125		
Kreizinger et al., 2013	Hungary	B12 (29)	2003–2010	E-test / mFrancis agar	0.38–1	0.75	3–8	6
Yesilyurt et al., 2011	Turkey	B bvII (39)	2009–2010	E-test / GCBA	0.094–0.25	0.25	0.75–1.5	1.5
Tomaso et al., 2005	Austria	B bvII (50)	1992–1998	E-test / CHAB	0.094–2	0.75	0.75–8	3
Ikäheimo et al., 2000	Finland	B (38)	NA	E-test / CHAHb	0.38–1.5	1	0.25–4	4
Kiliç et al., 2013	Turkey	B bvII (249)	2009–2012	E-test / CHA	0.094–0.38	0.25	0.5–2	1.5
Johansson et al., 2002	USA	A (8)	1996–2001	E-test / MHI + 1% IsoVx or CHAB	0.032–0.25		0.064–2	
		B (16)			0.016–0.125	0.064	0.064–1	0.25
Velinov et al., 2011	Bulgaria	B bvII (21)	NA	E-test / caMHB + 2% IsoVx	0.064–0.5	0.125	0.25–2	1
Tomaso et al., 2017	Germany	1B4, 3B6, 34B12 (128)	2005–2014	E-test / CHAB	0.047–2	1	0.75–8	4
All E-tests					0.016–2	0.064–1	0.064–8	0.25–6

Methods: BMD: broth microdilution technique; E-test: E-test strip method. Culture media: caMHB: cation-adjusted MHB; mThayer-Martin : modified Thayer-Martin (i.e., Thayer-Martin agar with hemoglobin and polyvitamins supplement); mFrancis agar: modified Francis agar (i.e., chocolate agar plate containing 1% glucose and 0.1% cysteine); GCBA: glucose/cysteine blood agar (i.e., brain heart infusion agar supplemented with 1% glucose monohydrate, 0.1% L-cysteine, and 9% chocolatized sheep blood); CHA: cysteine heart agar; CHAB: CHA + 10% sheep blood; CHAHb: CHA + 2% hemoglobin; IsoVx: IsoVitaleX™.

Tetracycline and doxycycline displayed MIC and MIC90 ranges lower than the CLSI susceptibility breakpoint (≤4 mg/L) for all *F. tularensis* strains tested (Table 3). Tigecycline displayed similar activity, with MIC and MIC90 ranges of ≤0.03–1 mg/L and 0.25–1 mg/L for the BMD method (Heine et al., 2017; Caspar et al., 2018) and 0.094–0.38 mg/L and 0.19–0.25 mg/L for the E-test method (Yesilyurt et al., 2011; Kreizinger et al., 2013).

**TABLE 3:** MICs of fluoroquinolones against *Francisella tularensis.*

References	*F. tularensis* strains			Method / medium	Doxycycline MICs (mg/L)		Tetracycline MICs (mg/L)		Ciprofloxacin MICs (mg/L)		Levofloxacin MICs (mg/L)	
	Country source	Type (number)	Isolated in		Range	MIC90	Range	MIC90	Range	MIC90	Range	MIC90
Origgi et al., 2014	Switzerland	B6 (19)	1996–2013	BMD / caMHB + 2% IsoVx			≤0.25	≤0.25	≤0.06	≤0.06		
		B12 (5)					≤0.25		≤0.06			
Georgi et al., 2012	Europe	B (69)	NA	BMD / caMHB + 2% IsoVx			≤0.125–2	1	≤0.031–0.125	0.063	≤0.031–0.125	0.063
	North America	A (7)					≤0.125–0.5		0.031–0.125			
Urich and Petersen, 2008	North America	A (92)	1974–2005	BMD / caMHB + 2% IsoVx	0.25–4	2	0.25–2	1	0.004–0.06	0.06	0.015–0.12	0.06
		B (77)			0.25–2	2	0.12–2	1	0.008–0.06	0.03	0.015–0.12	0.06
Heine et al., 2017	Variable	A (19), B (10)	1920–2002	BMD / caMHB + 2% IsoVx	≤0.03–4	0.25	≤0.03–1	0.25	0.015–0.25	0.25	0.008–0.25	0.06
Caspar et al., 2018	France	B6 (59)	2006–2017	BMD / caMHB + 2% IsoVx	0.125–0.25	0.25			0.016-0.06	0.03	0.016–0.06	0.06
All BMD					≤0.03–4	0.25–2	≤0.125–2	≤0.25–1	0.004–0.25	0.03–0.25	0.008–0.25	0.06
Johansson et al., 2000	Sweden	B (7)	1998	E-test / mThayer-Martin	0.25–0.5				0.008–0.015			
Hotta et al., 2013	Japan	B (36)	1926–1989	E-test/ Chocolate II agar	0.094–1.5				0.003–0.023	0.023		
Kreizinger et al., 2013	Hungary	B12 (29)	2003–2010	E-test / mFrancis agar	0.12–1.5	1	0.19–0.72	0.5	0.012–0.047	0.047	0.004–0.023	0.023
Yesilyurt et al., 2011	Turkey	B bvII (39)	2009–2010	E-test / GCBA			0.125–0.5	0.38	0.008–0.016	0.016	0.006–0.016	0.012
Tomaso et al., 2005	Austria	B bvII (50)	1992–1998	E-test / CHAB	0.38–3	2	0.125–0.75	0.75	0.004–0.125	0.032	0.008–0.047	0.032
Ikäheimo et al., 2000	Finland	B (38)	NA	E-test / CHAHb			0.094–0.5	0.38	0.008–0.023	0.016	0.008–0.023	0.016
Kiliç et al., 2013	Turkey	B bvII (249)	2009–2012	E-test / CHA	0.064–0.38	0.25	0.094–0.5	0.38	0.004–0.023	0.016	0.003–0.016	0.012
Johansson et al., 2002	USA	A (8)	1996–2001	E-test / MHI + 1% IsoVx or CHAB	0.125–2				0.016–0.064		0.016–0.064	
		B (16)							0.016–0.064	0.064	0.008–0.125	0.125
Velinov et al., 2011	Bulgaria	B bvII (21)	NA	E-test / caMHB + 2% IsoVx	0.25–4	2			0.002–0.06	0.047	0.016–0.125	0.094
Tomaso et al., 2017	Germany	1B4, 93B6, 34B12 (128)	2005–2014	E-test / CHAB	0.19–3	1.5	0.064–3	0.5	0.002–0.25	0.064		
All E-test					0.064–4	0.25–2	0.094–3	0.38–0.75	0.002–0.25	0.016–0.064	0.003–0.125	0.012–0.125

Methods: BMD: broth microdilution technique; E-test: E-test strip method. Culture media: caMHB: cation-adjusted MHB; mThayer-Martin : modified Thayer-Martin (i.e., Thayer-Martin agar with hemoglobin and polyvitamins supplement); mFrancis agar: modified Francis agar (i.e., chocolate agar plate containing 1% glucose and 0.1% cysteine); GCBA: glucose/cysteine blood agar (i.e., brain heart infusion agar supplemented with 1% glucose monohydrate, 0.1% L-cysteine, and 9% chocolatized sheep blood); CHA: cysteine heart agar; CHAB: CHA + 10% sheep blood; CHAHb: CHA + 2% hemoglobin; IsoVx: IsoVitaleX™.

The fluoroquinolones are the most effective antibiotics against *F. tularensis in vitro* (Table 3). All *F. tularensis* strains tested were susceptible to ciprofloxacin and levofloxacin according to CLSI susceptibility breakpoint (≤0.5 mg/L). Moxifloxacin displayed similar activity with MIC and MIC90 ranges of ≤ 0.004–0.25 mg/L and 0.06–0.125 mg/L for the BMD method (Heine et al., 2017; Caspar et al., 2018) and 0.012–0.125 mg/L and 0.032–0.125 mg/L for the E-test method (Johansson et al., 2002; Tomaso et al., 2005; Yesilyurt et al., 2011).

The activity of the macrolides depends on the *F. tularensis* clade (Table 4). Type B biovar (bv) II strains (currently clade B12) are naturally highly resistant to the macrolides, with erythromycin and azithromycin MICs > 256 mg/L. The other A and B clades display lower MICs to the macrolides although variables between studies. Overall, using the BMD method, erythromycin MIC and MIC90 ranges were 0.5–8 mg/L and 1–4 mg/L, respectively. Azithromycin was more effective, with MIC ranges of 0.125–2 mg/L. No macrolide CLSI susceptibility breakpoint is available for *F. tularensis*.

**TABLE 4:** MICs of macrolides, chloramphenicol, and rifampicin against *Francisella tularensis.*

References	*F. tularensis* strains			Method/medium	Erythromycin MICs (mg/L)		Chloramphenicol MICs (mg/L)		Rifampicin MICs (mg/L)	
	Country source	Type (number)	Isolated in		Range	MIC90	Range	MIC90	Range	MIC90
Origgi et al., 2014	Switzerland	B6 (19)	1996–2013	BMD / caMHB + 2% IsoVx	2–8	4	≤2	≤2		
		B12 (5)			>32	>32	≤2			
Georgi et al., 2012	Europe	B (69)	NA	BMD / caMHB + 2% IsoVx	1− > 16	> 16	1–4	2	≤0.5–2	
	North America	A (7)			ND	ND	≤0.5			
Urich and Petersen, 2008	North America	A (92)	1974–2005	BMD / caMHB + 2% IsoVx	0.5–4	2	0.5–4	2		
		B (77)			0.5–2	0.5	0.5–4	2		
Heine et al., 2017	Variable	A (19), B (10)	1920–2002	BMD / caMHB + 2% IsoVx			0.25–8	4	≤0.03–2	0.5
Caspar et al., 2018	France	B6 (59)	2006–2017	BMD / caMHB + 2% IsoVx	0.5–2	1	0.5–2	2	0.125–1	1
All BMD							0.25–8	≤2–4	≤0.003–2	0.5–1
BMD for B12 / bv II					>32	>32				
BMD for A + B6					0.5–8	1–4				
Johansson et al., 2000	Sweden	B (7)	1998	E-test / mThayer-Martin	>256		0.25		0.5	
Hotta et al., 2013	Japan	B (36)	1926–1989	E-test/ Chocolate II agar	0.094–1.5	1.5				
Kreizinger et al., 2013	Hungary	B12 (29)	2003–2010	E-test / mFrancis agar	>256	>256	0.5–1.5	1.5	0.5–2	1
Yesilyurt et al., 2011	Turkey	B bvII (39)	2009–2010	E-test / GCBA	>256	>256	0.094–0.25	0.25	0.25–1	0.75
Tomaso et al., 2005	Austria	B bvII (50)	1992–1998	E-test / CHAB	>256	>256	0.023–2	0.75	0.25–3	1.5
Ikäheimo et al., 2000	Finland	B (38)	NA	E-test / CHAHb			0.125–0.5	0.38	0.094–0.38	0.25
Kiliç et al., 2013	Turkey	B bvII (249), bv Japonica (1)	2009–2012	E-test / CHA	>256	>256	0.094–0.75	0.5	0.125–1	0.75
Johansson et al., 2002	USA	A (8)	1996–2001	E-test / MHI + 1% IsoVx or CHAB	0.125–1		0.5–1		0.25–2	
		B (16)			0.125–1		0.25–1	1	0.125–1	1
Velinov et al., 2011	Bulgaria	B bvII (21)	NA	E-test / caMHB + 2% IsoVx	>256	>256	1–4	2		
Tomaso et al., 2017	Germany	1B4, 93B6, 34B12	2005–2014	E-test / CHAB	0.5–8 ≥ 8		0.125–3	3		
All E-tests							0.023–4	0.25–3	0.094–3	0.25–1.5
E-tests for B12 / bvII					>256	>256				
E-tests for A + B6					0.125–8					

Methods: BMD: broth microdilution technique; E-test: E-test strip method. Culture media: caMHB: cation-adjusted MHB; mThayer-Martin : modified Thayer-Martin (i.e., Thayer-Martin agar with hemoglobin and polyvitamins supplement); mFrancis agar: modified Francis agar (i.e., chocolate agar plate containing 1% glucose and 0.1% cysteine); GCBA: glucose/cysteine blood agar (i.e., brain heart infusion agar supplemented with 1% glucose monohydrate, 0.1% L-cysteine, and 9% chocolatized sheep blood); CHA: cysteine heart agar; CHAB: CHA + 10% sheep blood; CHAHb: CHA + 2% hemoglobin; IsoVx: IsoVitaleX™.

Chloramphenicol and rifampicin are considered less effective against *F. tularensis*, although no CLSI breakpoint is available for this species (Table 4). Moreover, rapid resistance to rifampicin may occur through mutations in the RNA polymerase encoding gene (Bhatnagar et al., 1994; Zaw et al., 2018).

Trimethoprim-sulfamethoxazole (ratio 1:19) was poorly active against *F. tularensis*, with MICs ≥ 4/76 mg/L for 69 type B strains from Europe (Georgi et al., 2012) and MIC ranges of 0.25/4.75–8/152 mg/L (MIC90, 4/76 mg/L) for 19 type A and 10 type B strains of variable origin (Heine et al., 2017).

Although we did not find MICs for glycopeptides and polymyxins against *F. tularensis*, it is likely that this bacterium is resistant to both antibiotic classes since its isolation from environmental samples was obtained using a selective medium containing 4 mg/L of vancomycin and 8 x 10^4^ U/L of polymyxin B (Petersen et al., 2009).

#### 3.1.3 Bactericidal activity

A few studies have evaluated the bactericidal activity of antibiotics against *F. tularensis* by determining the MBCs (minimum antibiotic concentration allowing 3-log or greater reduction of a bacterial inoculum). For fluoroquinolones (ofloxacin and ciprofloxacin) MBCs were close to MICs demonstrating the high bactericidal activity of these antibiotics against *F. tularensis* (Maurin et al., 2000; Aloni-Grinstein et al., 2015). The MBC/MIC ratios ranged from 2 to 8 for gentamicin and streptomycin compatible with a bactericidal activity of these aminoglycosides, at least for the most susceptible strains (Maurin et al., 2000; Caspar et al., 2014; Aloni-Grinstein et al., 2015). Less favorable MBC/MIC ratios were found for doxycycline (i.e., from 4 up to 512) suggesting lack of bactericidal activity (Maurin et al., 2000; Aloni-Grinstein et al., 2015). One study found no bactericidal activity of doxycycline at concentrations up to 8 mg/L (Caspar et al., 2014). Therefore, tetracyclines should be considered mainly bacteriostatic against *F. tularensis*. Thiamphenicol, erythromycin, and trimethoprim-sulfamethoxazole displayed MBC/MIC ratios of 2, 4 and 16, respectively (Maurin et al., 2000; Aloni-Grinstein et al., 2015). However, due to their high MICs, these antibiotics can be considered weakly or non-bactericidal on *F. tularensis*. Rifampicin was more effective (MBC, 1 mg/L; MBC/MIC ratio of 2) (Maurin et al., 2000), but as previously mentioned this antibiotic must not be used as monotherapy.

### 3.2 Antibiotic activity in cell models

#### 3.2.1 Methods

The activity of antibiotics against intracellular *F. tularensis* was determined in variable eukaryotic cell systems, including murine macrophage-like cells (P388D1 and J774.1), Vero cells, A549 cells, and MRC5 cells (Maurin et al., 2000; Ahmad et al., 2010; Madrid et al., 2013; Sutera et al., 2014a; Aloni-Grinstein et al., 2015). The cell systems may also vary according to the bacterial inoculum used to infect cell monolayers and the multiplicity of infection (MOI, i.e., the ratio of bacteria to cells), the time antibiotics are added after cell infection, and the length antibiotic exposure of infected cells. The bacterial inoculum used to infect cells varied from 1.2 × 10^5^ CFU (Maurin et al., 2000) to 10^7^ CFU (Sutera et al., 2014a). MOIs varied from 1 to 3,000 (Ahmad et al., 2010; Madrid et al., 2013; Aloni-Grinstein et al., 2015). Antibiotics were usually added to culture supernatant after cell infection and removal of non-phagocytized bacteria (i.e., 1–3 h post-infection). Antibiotic activity was then evaluated after a short (15–24 h) (Ahmad et al., 2010; Madrid et al., 2013; Aloni-Grinstein et al., 2015) or long (3–5d) (Maurin et al., 2000; Sutera et al., 2014a) incubation period of antibiotic exposure of infected cells. The antimicrobial activity was deduced from: (1) changes in the intracellular bacterial load, using the CFU counting (Maurin et al., 2000) or a qPCR method (Aloni-Grinstein et al., 2015); or (2) changes in cell lysis rate caused by bacterial multiplication, after staining cell monolayers with a vital dye (Sutera et al., 2014a) or by measuring the release of lactate dehydrogenase (LDH) (Madrid et al., 2013). The minimum inhibitory extracellular concentration (MIEC) is the antibiotic concentration in the cell culture supernatant allowing complete inhibition of bacterial growth or cell lysis.

#### 3.2.2 Intracellular activity

Amoxicillin or ceftriaxone at 10 mg/L, or erythromycin, clarithromycin, or thiamphenicol at 4 mg/L, were ineffective against a *F. tularensis* in P388D1 cells (Maurin et al., 2000). Ciprofloxacin inhibited *F. tularensis* growth in P388D1 cells (at 1 mg/L) (Maurin et al., 2000) and in Vero cells (MIEC ranges of 0.06–0.25 mg/L) (Aloni-Grinstein et al., 2015). In MRC5 cells, ciprofloxacin, levofloxacin, and moxifloxacin were effective (MIEC of 0.064 mg/L, 0.125 mg/L, and 0.5 mg/L, respectively) (Sutera et al., 2014a). Gentamicin was found less effective (MIEC, 2 mg/L) in MRC5 cells and ineffective in Vero cells (MIEC > 20 mg/L) (Sutera et al., 2014a; Aloni-Grinstein et al., 2015). However, in a study allowing longer exposure of infected cells to gentamicin to facilitate its intracellular penetration, a bactericidal effect was observed at a concentration of 10 mg/L of this antibiotic (Maurin et al., 2000). Doxycycline displayed MIECs of 0.125–0.5 in Vero cells (Aloni-Grinstein et al., 2015) and 0.5 mg/L in MRC5 cells (Sutera et al., 2014a), and was also effective in P388D1 cells (Maurin et al., 2000). MIECs were 0.5 mg/L for rifampicin in MRC5 cells (Sutera et al., 2014a), and 1–2 mg/L for chloramphenicol in Vero cells (Aloni-Grinstein et al., 2015). Erythromycin displayed MIECs of 1–2 mg/L for two type B bvI isolates grown in MRC5 cells (Sutera et al., 2014a) and > 256 mg/L for the type B bvII LVS strain in Vero cells (Aloni-Grinstein et al., 2015). Azithromycin was bactericidal against the LVS strain at 5 mg/L in J774.1 cells, but only at 25 mg/L in human lung epithelial A549 cells, suggesting better penetration of this antibiotic in the former cells (Ahmad et al., 2010). Finally, the protection level of J774.1 cells infected with the SchuS4 strain was 100% for tetracycline at 50 μM (22 mg/L) and erythromycin at 50 μM (37 mg/L) and 92% for minocycline at 50 μM (23 mg/L) (Madrid et al., 2013).

Overall, data obtained in cell models are difficult to interpret because of methodological heterogeneity between studies. However, the fluoroquinolones had the strongest bactericidal activity against intracellular *F. tularensis*, gentamicin had a delayed bactericidal activity, and doxycycline was only bacteriostatic. Among other antibiotic classes, rifampicin displayed the lowest MIECs.

## 4 Antibiotic resistance in *F. tularensis*

### 4.1 Natural resistance

*Francisella tularensis* is naturally resistant to most beta-lactams, polymyxins, glycopeptides, sulfonamides, and trimethoprim (Caspar and Maurin, 2017; Kassinger and van Hoek, 2021). Two genes code for beta-lactamases, including Bla1 considered as a non-functional beta-lactamase (Antunes et al., 2012) and Bla2 (or FTU-1), a weak Ambler Class A carbapenemase inducing high-level resistance to penicillins but low-level resistance to cephalosporins and carbapenems (Bina et al., 2006). An AcrAB RND efflux system confers resistance to ampicillin, carbenicillin, and cefoperazone in the LVS strain (Bina et al., 2008). Natural high-level resistance to the macrolides in type B bv II (clade B12) strains is due to A2059C mutation (compared to other clades) in the three copies of the *rrl* gene encoding the 23S rRNA (Karlsson et al., 2016).

The *F. tularensis* genome contains genes that have been associated with antibiotic resistance in other bacterial species, but not in *Francisella* species. They include a tetracycline MFS-type multidrug transporter (Tet) (Biswas et al., 2008) and genes inducing colistin resistance by lipid A modification (Wang et al., 2007; Li et al., 2012). Some genes code for efflux pumps potentially associated with antibiotic resistance, including AcrAB-TolC for resistance to beta-lactams (Bina et al., 2008; Caspar and Maurin, 2017; Kassinger and van Hoek, 2021), aminoglycosides (especially, gentamicin and streptomycin) (Gil et al., 2006), and azithromycin (Ahmad et al., 2010); EmrA1 for resistance to streptomycin, neomycin, and tetracyclines (Ma et al., 2014); TolC (Gil et al., 2006) for resistance to tetracyclines; and SilC (Alqahtani et al., 2018) for resistance to nalidixic acid.

### 4.2 Acquired resistance to antibiotics

No acquired resistance to antibiotics has been reported so far in natural humans or animal strains of *F. tularensis* (Caspar and Maurin, 2017; Kassinger and van Hoek, 2021). *In vitro*, antibiotic-resistant mutants have been selected in the virulence attenuated LVS strain. Resistance to fluoroquinolones was mainly associated with mutations in the genes coding DNA gyrase (*gyrA* and *gyrB*) or topoisomerase IV (*parC* and *parE*) (La Scola et al., 2008; Sutera et al., 2014b,^2020^; Jaing et al., 2016; Caspar et al., 2017; Biot et al., 2020), and in genes coding for efflux pump of the MexH family RND transporter or AcrAB-TolC (Biot et al., 2020). Deletion of *fupA/B* gene, coding an iron-binding membrane protein, was also involved (Siebert et al., 2019). High-level erythromycin resistance was selected in type B bv I isolates due to mutations in the 23S rRNA gene (Gestin et al., 2010). Tetracycline resistance was obtained by introducing a plasmid containing the Tet repressor gene (TetR) (Pavlov et al., 1996; LoVullo et al., 2012; Sheshko et al., 2021). Streptomycin resistance was associated with mutations involving several genes, including *rpsL* gene coding the 30S ribosomal protein S12 (Biot et al., 2020). Rifampicin-resistance was related to mutations in *rpoB* gene, coding the subunit B of the RNA polymerase (Bhatnagar et al., 1994). Combined resistances to ciprofloxacin, doxycycline, and chloramphenicol were also selected in the LVS strain (Sutera et al., 2014b; Biot et al., 2020; Mehta et al., 2022).

## 5 Antibiotic activity in animal models

Tularemia animal models have included mice, rats, guinea pigs, rabbits, and non-human primates (NHP) (Stundick et al., 2013). The disease severity depends on the animal species, bacterial strain, and dose and route of infection. Mice usually develop a fatal infection when challenged with a low dose of type A (1–10 cfu) or type B (10–100 CFU) strain, including the LVS strain. Fischer 344 rats are more susceptible to type A infection than Sprague–Dawley rats, but both are resistant to LVS infection. Among NHP, African green monkey and Cynomolgus macaques are more susceptible to type A infection than Rhesus macaques (Glynn et al., 2015).

In mice infected with *F. tularensis*, results varied according to the bacterial strain inoculated (Schu S4 or LVS strain) and the antibiotic inoculation route, dose, duration, and time post-infection (p.i.) (Table 5). Mice infected with Schu S4 usually survived when treated 24 h p.i. at appropriate dosage of ciprofloxacin (Rotem et al., 2012; Barnes et al., 2021), levofloxacin (Klimpel et al., 2008; Peterson et al., 2010; Crane et al., 2012), and moxifloxacin (Piercy et al., 2005). Survival rates were lower when treatment was given orally compared to intravenously or intraperitoneally (Piercy et al., 2005; Steward et al., 2006), or delayed at 48 h or 72 h p.i. (Klimpel et al., 2008; Peterson et al., 2010; Crane et al., 2012; Rotem et al., 2012; Barnes et al., 2021). Levofloxacin and moxifloxacin were more effective than ciprofloxacin (Piercy et al., 2005; Steward et al., 2006; Peterson et al., 2010; Crane et al., 2012). Doxycycline was less effective than fluoroquinolones in Schu S4-infected mice (Rotem et al., 2012; Grossman et al., 2017). In contrast doxycycline was as effective as ciprofloxacin in LVS-infected mice (Rotem et al., 2012). These studies also demonstrated the superiority of finafloxacin compared to ciprofloxacin (Barnes et al., 2021), and the fluorocycline TP-271 compared to doxycycline (Grossman et al., 2017). Several studies have reported the superiority of liposomal ciprofloxacin compared to the free form of this antibiotic (Hamblin et al., 2014). Recently, ceftobiprole medocaril, a new cephalosporin, has been reported as effective as levofloxacin to treat tularemia pneumonia caused by Schu S4 strain in Fischer 34 rats (Hahn et al., 2023).

**TABLE 5:** Survival rates in *F. tularensis*-infected mice treated with antibiotics compared to untreated animals (100% death rate).

References	Animal	*Francisella* infection		Antibiotic treatment				Survival rate
		Strain	Route	Drugs	Dosage per day, route	Number of days	Time post- infection	
Hahn et al., 2023	Fischer 344 rats	Schu S4	Aerosol, 1000 CFU	Ceftobiprole medocaril	145 mg/kg tid, iv	14	72 h	92%
				Levofloxacin	50 mg/kg, bid	14	72 h	92%
Barnes et al., 2021	Balb/c mice	Schu S4	Aerosol, ∼300 CFU	Finafloxacin	23.1 mg/kg tid, po	3	24 h	100%
						7		100%
						3	72 h	0% delayed death
						7		50%
				Ciprofloxacin	30 mg/kg, bid, ip	3	24 h	100%
						7		100%
						3	72 h	0% more rapid death
						7		10%
Grossman et al., 2017	mice	Schu S4	Nasal, 91–283 CFU	TP-271 fluorocycline	3 mg/kg, ip	21	24 h	80% at d21*, 63% at d37**
							72 h	89% at d21, 100% at d37
					6 mg/kg, ip	21	24 h	100% at d21, 100% at d37
							72 h	100% at d21, 89% at d37
					12 mg/kg, ip	21	24 h	100% at d21, 80% at d37
							72 h	100% at d21, 100% at d37
					18 mg/kg, ip	21	24 h	100% at d21, 100% at d37
							72 h	100% at d21, 100% at d37
				doxycycline	40 mg/kg, bid, ip	21	24 h	84% at d21, 88% at d37
							72 h	100% at d21, 22% at d37
Crane et al., 2012	C57 Bl/6 mice	Schu S4	Intranasal, 50 CFU,	levofloxacin	40 mg/kg	14	d1, d2, or d3	100% at d30
					5 mg/kg	14	d1, d2, or d3	100% at d30 for d1 and d2, 60% for d3 pi treatment
Peterson et al., 2010	BALB/c mice	Schu S4	3 LD50 (1.7 x 10^2^ CFU) intranasal	Levofloxacin	0.1, 0.5, or 1 mg/kg, ip	13	24 h	50%, 100%, 90%
					5, 10 mg/kg, ip	13	24 h	100%
					40 mg/kg, ip	13	72 h, 96 h, 120 h	100%, 80%, 0%
Klimpel et al., 2008	BALB/c mice	Schu S4	Intranasal, ∼100 CFU, (3 x LD50) intranasal	Levofloxacin	6.25, 12.5, 25, or 50 mg/kg, ip	13	24 h	100%
					40 mg/kg	13	48 h, 72 h, 96 h, or 120 h	100%, 100%, 80%, 0%
Steward et al., 2006	BALB/c mice	Schu S4	1.5 × 10^4^ CFU nose-only aerosol	Ciprofloxacin	100 mg/kg, po, bid	14	6 h, 24 h, 48 h	0% at 42 p.i.
				Moxifloxacin			6 h, 24 h, 48 h	53%, 12%, 35% at 42 p.i.
				Gatifloxacin			6 h, 24 h, 48 h	53%, 41%, 65% at 42 p.i.
Piercy et al., 2005	BALB/c mice	Schu S4	Subcutaneous, 10^6^ CFU	Ciprofloxacin	100 mg/kg, tid, po	14	6 h, 24 h, 48 h	94%/67%/0%
				Gatifloxacin	100 mg/kg, tid, po	14	6 h, 24 h, 48 h	100%/96%/84%
				Moxifloxacin	100 mg/kg, tid, po	14	6 h, 24 h, 48 h	100%/100%/62%
Rotem et al., 2012	BALB/c mice	Schu S4	10^2^ CFU (100 x LD50), intranasal	Ciprofloxacin	50 mg/kg, bid, ip	7	24 h, 48 h, 72 h	100%, 100%, 70%
				Doxycycline	40 mg/kg, bid, ip	14	24 h, 48 h, 72 h	90%, 30%, 0%
				Ciprofloxacin	50 mg/kg, bid, ip	10	72 h	100%, no relapse
				Doxycycline	40 mg/kg, bid, ip	21	72 h	10%, bacteria detectable 2 days after treatment stop (10^4^ cfu)
		LVS	10^5^ fu (100 x LD50), intranasal	Ciprofloxacin	50 mg/kg, bid, ip	7	24 h, 48 h, 72 h	100%, 100%, 100%
				Doxycycline	40 mg/kg, bid, ip	14	24 h, 48 h, 72 h	100%, 100%, 100%
Di Ninno et al., 1993	BALB/c mice	LVS	10 x LD50, iv or intranasal	Ciprofloxacin	1 mg iv	1	1d, 2d, 3d, 7d	0%, 25%, 0%, 12%
					1 mg in			50%, 0%, 25%, 0%
				Liposomal ciprofloxacin	1 mg iv	1	1d, 2d, 3d, 7d	75%, 88%, 0%, 0%
					1 mg in			83%, 100%, 63%, 50%

Antibiotic administration route, po: per os, in: intranasal, ip: intraperitoneal, iv: intravenous, pi: post-infection.

*Percentage of animals that survived at the end of treatment (d21);

**Percentage of animals among the survivors that survived the following 16 days (d37).

## 6 Tularemia treatment in humans

### 6.1 General comments

The following paragraphs summarize literature data on treatment efficacy in tularemia patients according to different clinical situations. Although some patients are likely redundant across different publications, this review includes 2,482 tularemia cases, corresponding to 1,174 (47.3%) men, 1,173 (47.3%) women, while for 135 (5.8%) patients the sex was not specified. The pediatric population represents 10.6% of these cases. All tularemia cases were reported as proven or probable. Treatment data were not consistently reported between studies. Treatment durations were around 7–10 days for aminoglycosides and 2–3 weeks for tetracyclines and fluoroquinolones. However, the total duration of antibiotic treatment was often not specified, particularly for patients receiving several antibiotics either alternately or concomitantly. We considered the treatment failure and relapse rates reported by the authors without modification. Treatment failures and relapses usually corresponded to the absence of clinical improvement during or after appropriate antibiotic treatment and reappearance of clinical symptoms after a marked improvement or even an apparent cure, respectively. In both cases, a new antibiotic treatment was administered often combined with a surgical procedure.

### 6.2 Acute, life-threatening diseases

Tularemia can manifest as acute diseases with spontaneous mortality rates up to 30% for type A strains, in North America (Dennis et al., 2001). These infections usually correspond to the typhoidal and pneumonic forms (Dennis et al., 2001; Feldman et al., 2001; Matyas et al., 2007). A World Health Organization group of consultants reported in 1970 that aerosol delivery of a virulent strain of *F. tularensis* (people inhaling at least 25 bacteria) would infect half of the exposed population, with a death rates of about 25% in untreated people and 1% in people receiving appropriate antibiotic prophylaxis within 48 h post-exposure (World Health Organization, 1970). Current treatment and prophylactic recommendations are well adapted to manage these acute infections (Dennis et al., 2001; Bossi et al., 2004). Gentamicin can be used in adults, including pregnant women, and children. Fatal infections, however, can occur despite appropriate treatment (Feldman et al., 2001).

### 6.3 Classical forms of tularemia in adults

#### 6.3.1 Ulceroglandular and glandular tularemia

Table 6 summarizes data from tularemia case series with a predominance of the UG or GL forms. Many other sporadic UG or GL tularemia cases have been reported in several countries but with similar findings than those reported in case series (Pérez-Castrillón et al., 2001; Jackson et al., 2012; Sobolewska-Pilarczyk et al., 2014; Boone et al., 2015; Formińska et al., 2015; Calin et al., 2017; Pekova et al., 2017; Whitten et al., 2017; Haulrig et al., 2020; Kubiliute et al., 2021). The UG and GL forms are predominant worldwide, except in some countries such as Turkey (see below).

**TABLE 6:** Antibiotic efficacy in tularemia patients with predominant UG or GL forms.

References	Country	Patients’ number, male/ female, age range or mean	Number or % of clinical forms	Antibiotics administered (number or % of patients)	Lymph node suppuration and surgery (number, % of patients)	Failures, relapses, and complications (number, % of patients)
Evans et al., 1985	USA	88, 69m/19w, 2–82 years	66 UG, 22 TY	str, gen, tet, chl, or comb	NA	Relapses, Str (2/30,7%), gen (2/6), tet (3/6), chl (3/5), two deaths
Pérez-Castrillón et al., 2001	Spain	142, 53m/89w, 14–82 years, mean age 52 +/- 14 years	87 UG, 13 GL, 29 TY, 6 OG, 5 PN, 2 OP	Initial treatment: Str (94), cip (22), dox (14), others (6)	NA	Failures after initial treatment: Str (23.4%), cip (4.5%), dox (42.8%)
Weber et al., 2012	USA	121, 79m/42w women, 6 months–92 years	45 UG, 30 GL, 14 PN, 12 TY, 4 OG, 2 OP	tet (49%), amg (47%), flq (41%)	Surgery, (15, 12.4%)	Hospitalization, 58%; one death
Maurin et al., 2011	France	101, 55m/46w, mean age 51.7 years	34 UG, 24 GL, 17 OP, 4 OG, 10 PN, 9 TY, 3 comb	cip (18), lev (2), mox (1), dox (25), gen (1), comb (9)	NA	Relapses, (39/101, 38.6%); hospitalization (29.7%); one death
Darmon-Curti et al., 2020	France	177, 123m/54w, mean age 47.4 years	34.4% UG, 27.1% GL, 18% PN, 7.9% TY, 5% OP, 2.3% OG	dox, flq	Suppuration, (39, 22%); drainage, (31, 40.3%); excision, (47, 26.5%)	Failures, dox (9.9%), flq (33.3%); hospitalization (53.4%)
Kızıl et al., 2012	Turkey	19, 7m/12w, 4–75y, mean age 43.3 years	12 GL, 1 OG, 6 OP	str (8), cip (5), dox (3), others (3) for 11–90 days	Suppuration, (15, 78.9%), surgery, (11, 57.9%)	NA

str: streptomycin, gen: gentamicin, amg: aminoglycoside, tet: tetracycline, dox: doxycycline, flq: fluoroquinolone, cip: ciprofloxacin, lev: levofloxacin, mox: moxifloxacin, chl: chloramphenicol, comb: combined antibiotics. Tularemia forms: ulceroglandular (UG), glandular (GL), oropharyngeal (OP), oculoglandular (OG), pneumonic (PN), and typhoidal (TY).

Beta-lactams (penicillin, amoxicillin, amoxicillin-clavulanate, cefuroxime, cefotaxime, ceftriaxone), macrolides (clarithromycin, clindamycin), and antituberculosis drugs often given as empirical treatments in UG and GL tularemia patients were ineffective (Maurin et al., 2011; Jackson et al., 2012; Sobolewska-Pilarczyk et al., 2014; Boone et al., 2015; Formińska et al., 2015; Pekova et al., 2017; Whitten et al., 2017; Haulrig et al., 2020; Kubiliute et al., 2021). Overall, gentamicin, ciprofloxacin (as well as levofloxacin and moxifloxacin) and doxycycline were the most successful treatments. However, several courses of antibiotic therapy were often prescribed because of poor clinical response and chronic evolution especially in the approximately 30% of UG or GL tularemia patients suffering from lymph node suppuration. The antibiotic therapy was adapted empirically according to the clinical and radiological findings. Treatment failure and relapse rates were usually high but variable between studies [e.g., 21.3% in Evans et al. (1985) and 38.6% in Maurin et al. (2011)]. Relapses were more frequent in patients with a significant treatment delay. For example, in the case series from Missouri (USA) (Weber et al., 2012), the mean treatment delay was 13 days (range, 0–82 days), but 21 days for patients evolving to lymph node suppuration. In a French series (Darmon-Curti et al., 2020), treatment delay was significantly longer (*p* = 0.006) for patients requiring surgery (mean, 52.1 days; range, 7–175 days) compared to those cured without surgery (mean, 32.1 days; range 1–87 days). In some studies, failure rates were higher for doxycycline versus ciprofloxacin (i.e., 4.5 versus 42.8%) (Pérez-Castrillón et al., 2001). Drainage or removal of suppurated lymph nodes were needed for cure in 12.4% up to 57.9% of patients (Maurin et al., 2011; Kızıl et al., 2012; Weber et al., 2012; Boone et al., 2015; Formińska et al., 2015). Many patients (29.7–58%) required hospitalization because of disease severity or more frequently the need for surgical intervention (Maurin et al., 2011; Weber et al., 2012; Darmon-Curti et al., 2020). However, severe cases requiring hospitalization are more likely to be reported than milder cases and thus might be overestimated in the literature. In some patients, complications occurred while under appropriate antibiotic therapy, including continued enlargement of the infected lymph nodes, involvement of new lymph nodes, regional and systemic complications (Jackson et al., 2012; Kızıl et al., 2012; Weber et al., 2012; Formińska et al., 2015; Calin et al., 2017; Haulrig et al., 2020; Kubiliute et al., 2021). A few patients with UG or GL forms died (2/418, 0.48% for the above sporadic cases and case series). Death usually occurred in patients with underlying health condition, e.g., a cardiovascular disease (Weber et al., 2012). Altogether, the above data emphasizes the need for specific medical and surgical treatment recommendations for patients suffering from UG or GL forms of tularemia, especially when experiencing lymph node suppuration.

#### 6.3.2 Oropharyngeal tularemia

At onset, OP tularemia looks like streptococcal pharyngitis leading to beta-lactam treatment. Due to oral route of infection, complications include lymph node suppuration, parapharyngeal abscess, digestive involvement, and systemic diseases. Large OP tularemia case series have been reported in Turkey where these infections predominate and occur as outbreaks mainly related to the consumption of contaminated water (Meric et al., 2008; Ulu-Kilic et al., 2013; Erdem et al., 2014; Gozel et al., 2014; Table 7). Many other small series or sporadic OP tularemia cases have been reported involving more than 200 patients (Arikan et al., 2003; Chitadze et al., 2009; Sencan et al., 2009; Komitova et al., 2010; Kızıl et al., 2012; Dentan et al., 2013; Eren Gok et al., 2014; Karakas et al., 2014; Uzun et al., 2015; Esmaeili et al., 2021; Binay et al., 2023).

**TABLE 7:** Antibiotic efficacy in tularemia patients with predominant OP forms.

References	Country	Patients’ number, male/ female, age range or mean	Number or % of clinical forms	Antibiotics administered (number or % of patients)	Lymph node suppuration and surgery (number, % of patients)	Failures, relapses, and complications (number, % of patients)
Erdem et al., 2014	Turkey	1,034, 446m/588w, mean age 41y	85.3% OP, 13.1% GL, 10.1% UG, some combined forms	Str (28%), gen (8%), dox (12%), cip (18%), combined or sequential (30.3%)	Suppuration (41.3%); fine-needle aspiration (50%), drainage (12%), and excision (3%).	Failures (48%), relapses (24, 2.3%) with antibiotics plus surgery, including str (7/291, 2.4%), gen (2/85, 2.3%), dox (1/127, 0.78%), cip (2/188, 1.06%), comb (10/299, 3.34%)
Meric et al., 2008	Turkey	145, 59m/86w	100% OP			Failures (55, 37.9%), including amg (14, 9.6%), flq (14, 9.6%), and tet (27, 18.6%)
Ulu-Kilic et al., 2013	Turkey	139, 55m/84w, mean age 43y	74% OP, 15.8% GL	Str (40), gen (6), cip (79)	Aspiration or drainage (51, 39%)	Failures (43, 30.9 %)
Gozel et al., 2014	Turkey	68, 31m/37w	100% OP	Cip (25), str (18), dox (2), str + dox (23)	Drainage (24, 35.3%)	Failures, 6 (8.8%)
Binay et al., 2023	Turkey	16, 6m/10f, mean age 39.4y	81.3% OP, 18.7% GL	Mox (9), str (1), dox (1), combined (5)	Drainage (5, 31.2%)	None

str: streptomycin, gen: gentamicin, dox: doxycycline, cip: ciprofloxacin. Tularemia forms: ulceroglandular (UG), glandular (GL), oropharyngeal (OP), oculoglandular (OG), pneumonic (PN), and typhoidal (TY).

Patients with the OP form of tularemia did not respond to empirical treatment with beta-lactams, macrolides or antituberculosis drugs (Arikan et al., 2003; Sencan et al., 2009; Willke et al., 2009; Cağlı et al., 2011; Erdem et al., 2014; Binay et al., 2023). Adapted antibiotic treatments most often included an aminoglycoside, ciprofloxacin, or doxycycline in descending order of prescription frequency. These antibiotics were often combined or given sequentially. For example, among antibiotic treatments given to 1,034 tularemia patients (including 85.3% OP) in Turkey, an aminoglycoside was prescribed in 36% of patients (28% for streptomycin), a fluoroquinolone in 20% (18% for ciprofloxacin), a tetracycline in 12.5% (12% for doxycycline), and a combined treatment in 28.8% (Erdem et al., 2014). Global failure rates were high but varied between studies, e.g., 30.9% for Ulu-Kilic et al. (2013), 37.9% for Meric et al. (2008), and 50.4% for Erdem et al. (2014). Relapse rates also varied between studies, e.g., 9.09% in Cağlı et al. (2011) and 78.9% in Kızıl et al. (2012). These variations might reflect differences in the recruited patients. Failures and relapses were less frequent when antibiotics were administered within 2–3 weeks of symptoms onset (Meric et al., 2008; Komitova et al., 2010). Doxycycline was associated with higher failure and relapse rates then aminoglycosides and ciprofloxacin (Meric et al., 2008; Erdem et al., 2014). For example, Meric et al. (2008) reported a global failure rate of 37.9% among 145 OP cases, including 9.6% each for aminoglycosides and fluoroquinolones, but 18.6% for tetracyclines. Lymph node suppuration was frequent [e.g., 41.2% in Erdem et al. (2014)]. Surgical drainage or excision of suppurated lymph nodes were required in about 31% (Karakas et al., 2014; Binay et al., 2023) up to 69.7% (Cağlı et al., 2011) of patients with OP tularemia. Among 254 patients (including 217 OP forms) reported as sporadic cases and in small case series (see references above), lymph node resection or drainage was performed in 51.2% (130/254) of patients. Lymph node excision was found more effective than drainage or needle aspiration of lymph node suppuration to prevent relapses (Kızıl et al., 2012). Although OP tularemia can evolve over several months and occasionally lead to severe complications, no fatal cases were reported in the above case series and sporadic cases.

#### 6.3.3 Oculoglandular tularemia

Only sporadic OG tularemia cases have been reported (Pärssinen and Rummukainen, 1997; Komitova et al., 2010; Mahy et al., 2011; Altuntas et al., 2012; Eren Gok et al., 2014; Darmon-Curti et al., 2020). Patients usually suffered from painful unilateral conjunctivitis with lymphadenopathy (mainly in the periauricular and submandibular areas), which defines the Parinaud’s oculoglandular syndrome (Weber et al., 2012; Eren Gok et al., 2014). Diagnosis was often delayed because *F. tularensis* is only a rare cause of this syndrome and therefore evoked in the second line. Beta-lactams (e.g., amoxicillin-clavulanate or ampicillin-sulbactam) and macrolides (e.g., pristinamycin) were ineffective (Mahy et al., 2011; Altuntas et al., 2012; Darmon-Curti et al., 2020). Specific antibiotic treatment was usually an aminoglycoside or a fluoroquinolone for 10–14 days, or doxycycline for 2–3 weeks, or an antibiotic combination. Eren Gok et al. (2014) advocated adding a topical antibiotic treatment, including ciprofloxacin drops or tobramycin drops and ointment for 7 days. In most patients, ocular symptoms regressed under appropriate treatment within 7–10 days. Four (22.2%) of the 18 above cases required drainage or excision of suppurated lymph nodes. All cases recovered without sequelae.

The same treatment options were used for treatment of keratitis and dacryocystitis (Steinemann et al., 1999; Celik et al., 2013; Köse and Hoşal, 2019). Three-weeks treatment with doxycycline was effective to treat posterior uveitis in a 52-year-old man, but with loss of vision of the involved eye (Terrada et al., 2016). Although treatment of *F. tularensis* intraocular infection is not standardized, it should be emphasized that ciprofloxacin intravenously has more favorable intraocular pharmacokinetics properties than doxycycline and gentamicin (Kulshrestha et al., 1981; Mounier et al., 1988; Fiscella et al., 1998; Morlet et al., 2000).

#### 6.3.4 Pneumonic tularemia

Pneumonic tularemia is most often of mild to moderate severity (see above for severe type A pneumonia) (Table 8). Type B pneumonia typically have a prolonged course, with intermittent fever, mild respiratory symptoms, progressive weight loss, and development of mediastinal or hilar lymphadenopathy. Diagnosis is difficult and often delayed because of poor specificity of clinical and radiological findings. It should be suspected when pulmonary hypermetabolic lymph nodes are found on FDG-PET/CT scan in a clinical and epidemiological context compatible with tularemia (Martinet et al., 2021).

**TABLE 8:** Antibiotic efficacy in tularemia patients with PN forms.

References	Country	Patients’ number, male/ female, age range or mean in years (y)	Antibiotics administered (number or % of patients)	Failures, relapses, and complications (number, % of patients)
Matyas et al., 2007	USA	57 (m33y, f24y, m49y)	Gen + dox	One failure (lung consolidation, pleural effusion, and hilar lymphadenopathy) then recovery after a 2nd course of gen
Väyrynen et al., 2017	Finland	58, 45m/13f, 31–76 years, mean age 56y	flq (77.6%), tet (15.5%), amg (6.9%), comb (77.6%)	No relapse. Two patients required ICU admission
Fachinger et al., 2015	Switzerland	4 m, 40–62y	Cip 3wks; dox 3 weeks, relapse, then gen 1wk followed by cip 2 weeks; cip 3 weeks; and lev plus ceftriaxone 10d, relapse, cip	Two patients relapsed, one required ICU admission
Centers for Disease Control and Prevention (CDC), 2009	USA	26 (among 190 tularemia cases)	Tet, amg, flq, comb	Empyema requiring thoracotomy and decortication (2), and sepsis (2). Three patients required ICU admission
Darmon-Curti et al., 2020	France	32 (among 177 tularemia cases)	Tet, amg, flq, comb, no appropriate treatment in four patients	Complications: splenic hematoma, endocarditis, pericarditis, myocarditis
Rimawi et al., 2014	USA	2 (m62y, m61y)	Cip 2 weeks	ICU admission and ventilation for respiratory distress in both patients

gen: gentamicin, amg: aminoglycoside, tet: tetracycline, dox: doxycycline, flq: fluoroquinolone, cip: ciprofloxacin, lev: levofloxacin, comb: combined antibiotics. Tularemia forms: ulceroglandular (UG), glandular (GL), oropharyngeal (OP), oculoglandular (OG), pneumonic (PN), and typhoidal (TY).

Pneumonic tularemia has been reported in case series (Centers for Disease Control and Prevention (CDC), 2009; Väyrynen et al., 2017; Darmon-Curti et al., 2020) or as sporadic cases (Matyas et al., 2007; Fritzsch and Splettstoesser, 2010; Mahy et al., 2011; Rimawi et al., 2014; Fachinger et al., 2015). For the above three case series, representing 116 PN cases, no death was reported. Most patients had respiratory symptoms and 50% up to 90% had abnormal lung radiological findings, including frequent hilar or mediastinal lymphadenopathy. Väyrynen et al. (2017) reported a delay in hospital admission of 1–45 days (mean 9.9 days) and a hospital stay of 0–24 days (mean, 8.6 days). Among 84 patients from two series, five patients (5.9%) required admission to an intensive care unit due to respiratory distress [ Centers for Disease Control and Prevention (CDC), 2009; Väyrynen et al., 2017]. In these series from the USA and Finland, respectively, a fluoroquinolone was administered in 41 and 77.6% of patients, a tetracycline in 49 and 15.5%, an aminoglycoside in 47 and 6.9%, and these antibiotics were combined in most patients. Complications included sepsis, pleural effusion and empyema, endocarditis, pericarditis, and splenic hematoma [ Centers for Disease Control and Prevention (CDC), 2009; Väyrynen et al., 2017; Darmon-Curti et al., 2020].

When considering case series and sporadic cases, PN tularemia most frequently occurred in male patients (Matyas et al., 2007; Fritzsch and Splettstoesser, 2010; Rimawi et al., 2014; Fachinger et al., 2015; Ughetto et al., 2015; Väyrynen et al., 2017, 2017). Empirical treatment with beta-lactams (sultamicillin, ceftriaxone) or macrolides (azithromycin, clindamycin) was ineffective. The antibiotic treatment varied depending on the clinical presentation (mode of onset, severity), underlying medical condition of the patient, and type of infection (A versus B). For type A infections, gentamicin was the first-line treatment, occasionally combined with doxycycline or a fluoroquinolone (Matyas et al., 2007). However, ciprofloxacin alone was successful in some patients with severe pneumonia (Rimawi et al., 2014). Doxycycline alone, often administered upon suspicion of Rocky Mountain Spotted Fever, ehrlichiosis, or Lyme disease was less effective (Matyas et al., 2007). Patients with a prolonged course of type B pneumonia recovered after receiving gentamicin, ciprofloxacin, or a combination of both (Fritzsch and Splettstoesser, 2010; Mahy et al., 2011; Fachinger et al., 2015). Doxycycline administered for 3 weeks was reported as either effective (Darmon-Curti et al., 2020) or poorly effective (Fachinger et al., 2015). In a few cases, patients recovered while receiving no appropriate antibiotic therapy (Ughetto et al., 2015; Darmon-Curti et al., 2020). Surgery was occasionally required for excision of mediastinal or hilar lymph nodes, or empyema or pleural effusion drainage [ Centers for Disease Control and Prevention (CDC), 2009; Rimawi et al., 2014; Fachinger et al., 2015; Darmon-Curti et al., 2020].

#### 6.3.5 Typhoidal tularemia

Typhoidal tularemia is often a severe life-threatening disease (see above) but can also correspond to less severe clinical manifestations, in particular for type B infections (Han et al., 2004; Maurin et al., 2011; Nakamura et al., 2018). This form has been often reported in patients with underlying medical conditions, especially immunosuppression (Han et al., 2004; Maurin et al., 2011; Nakamura et al., 2018). Although tularemia is rarely fatal, a large proportion of deaths occur in patients with a TY form (Han et al., 2004; Maurin et al., 2011; Nakamura et al., 2018). Treatment data are scarce because TY forms are often mixed with other forms in case series (Hofinger et al., 2009; Maurin et al., 2011; Weber et al., 2012; Darmon-Curti et al., 2020). Whatever the severity of the disease, most patients have *F. tularensis* bacteremia and should receive an aminoglycoside (gentamicin or streptomycin, for 10–14 days) as a first-line antibiotic (Dennis et al., 2001; Bossi et al., 2004). We previously suggested combining an aminoglycoside with a fluoroquinolone in patients with severe tularemia for rapid extracellular and intracellular efficacy because the intracellular penetration of aminoglycosides is slow (Caspar and Maurin, 2017), but the superiority of combined antibiotic therapy compared to monotherapy is not established. Fortunately, empirical treatment in patients with TY tularemia often combined a beta-lactam (amoxicillin clavulanate, cefoperazone, or imipenem) with an antibiotic active against *F. tularensis* such as an aminoglycoside or a fluoroquinolone (Han et al., 2004; Maurin et al., 2011; Nakamura et al., 2018). When an aminoglycoside was used, an oral relay with a fluoroquinolone or a tetracycline for 2 weeks was often considered. It is notable that relapses usually do not occur in patients with TY tularemia receiving appropriate antibiotic therapy.

### 6.4 Complications

#### 6.4.1 Skin rashes

Skin involvement in tularemia patients include primary lesions such as the inoculation eschars and secondary lesions (tularemids) that occur in approximately 10–20% of cases (Syrjälä et al., 1984; Evans et al., 1985; Cerný, 1994; Meric et al., 2008; Ulu-Kilic et al., 2013; Erdem et al., 2014; Gozel et al., 2014; Şenel et al., 2015; Polat et al., 2018). Secondary lesions might not be considered as complications, but frequently occur in patients with systemic infections (Syrjälä et al., 1984; Cerný, 1994; Polat et al., 2018). They include skin eruptions in 3–4% of patients (Erdem et al., 2014; Şenel et al., 2015; Polat et al., 2018), mainly of the macular or maculopapular type, or vesicular, vesiculopapular, or pustular types. Erythema multiforme has been reported in 2.8–11.3% of patients (Erdem et al., 2014; Şenel et al., 2015), and erythema nodosum in 2.6–13% of cases and mainly in women (Syrjälä et al., 1984; Akdiş et al., 1993; Erdem et al., 2014; Polat et al., 2018). Other skin manifestations include the Sweet syndrome [6.5% of 168 cases in Polat et al. (2018), urticaria (1.2–3.3% of cases (Şenel et al., 2015; Polat et al., 2018)], acne-like rash, Henoch-Schönlein purpura, and dermatitis (Syrjälä et al., 1984; Erdem et al., 2014; Şenel et al., 2015; Polat et al., 2018). Skin lesions can be combined (Polat et al., 2018). The skin lesions usually require no specific treatment and disappear quickly (a few days to 2–3 weeks) after the administration of streptomycin, gentamicin, doxycycline or a fluoroquinolone (Akdiş et al., 1993; Erdem et al., 2014; Marquart and Clifford, 2015; Şenel et al., 2015; Holland and Michelow, 2016).

#### 6.4.2 Soft tissue infections

Soft tissue infections in tularemia patients are rare and mainly include cellulitis and subcutaneous abscesses (Junkins and Snyder, 2011; Şenel et al., 2015; Arslan et al., 2016; Whitsell and Becker, 2020; Kreutzmann et al., 2021). They may occur through direct inoculation or hematogenous spread of *F. tularensis* to the skin or extension of a lymph node suppuration to the adjacent tissue.

A 49-year-old woman with facial cellulitis after a scalp tick bite was treated with amoxicillin-clavulanate plus gentamicin, then doxycycline plus ceftriaxone due to suspicion of Lyme disease (Arslan et al., 2016). After treatment failure, streptomycin was given for about 1 week for suspected tularemia with full recovery. A hand abscess with axillary and epitrochlear lymphadenopathy developed after a cat bite in a 66-year-old woman despite pasteurellosis prophylaxis with amoxicillin-clavulanate (Whitsell and Becker, 2020). She was cured after surgical drainage of the abscess and doxycycline treatment for 1 month. A 15-year-old boy developed OG tularemia with submandibular lymph node infection extending to the parotid gland and masseter muscle (Kosker et al., 2013). He was cured after suppurated lymph node excision and treatment with streptomycin (10 days) and tetracycline (8 weeks). A 60-year-old man with OG tularemia and orbital cellulitis did not improve under sulbactam-ampicillin treatment (Kreutzmann et al., 2021). He was cured after excision of suppurated cervical lymph nodes and treatment with ciprofloxacin relayed by doxycycline because of side effects.

#### 6.4.3 Meningitis and encephalitis

Approximately twenty cases of tularemia meningitis have been reported worldwide, mostly in the United States (Hofinger et al., 2009; Venkatesan et al., 2020). It was usually an inaugural manifestation of tularemia and diagnosis was based on *F. tularensis* isolation from the cerebrospinal fluid (CSF) (Gangat, 2007; Hofinger et al., 2009; Contentin et al., 2011; Venkatesan et al., 2020). This disease is spontaneously fatal (Venkatesan et al., 2020) and empirical treatments for meningitis (e.g., ceftriaxone, amoxicillin, vancomycin, and acyclovir) are ineffective (Weiner et al., 2004; Hofinger et al., 2009; Contentin et al., 2011; Venkatesan et al., 2020). Brain involvement has been rarely reported, including rhombencephalitis (Barbaz et al., 2013), meningitis with cerebral microabscesses (Gangat, 2007), and central nervous system vasculitis (Çoban et al., 2021).

Streptomycin, gentamicin, doxycycline, ciprofloxacin and chloramphenicol have been most frequently used for treating tularemia meningitis patients (Lovell et al., 1986; Hofinger et al., 2009; Contentin et al., 2011; Venkatesan et al., 2020; Ducatez et al., 2022). These antibiotics were often administered in combination or successively for a total duration of 2 to 8 weeks (Lovell et al., 1986; Hofinger et al., 2009; Contentin et al., 2011; Venkatesan et al., 2020; Ducatez et al., 2022). A 68-year-old man with tularemia meningitis was cured after receiving thiamphenicol for 6 days, then gentamicin for 18 days, then ciprofloxacin for 4 weeks (Contentin et al., 2011). In contrast, a 64-year-old man was cured after only 2 weeks of ciprofloxacin. Although tularemia meningitis treatment is not standardized, most cases occurring over the past two decades have recovered without sequelae while receiving variable antibiotic treatments (Gangat, 2007; Hofinger et al., 2009; Contentin et al., 2011; Venkatesan et al., 2020; Ducatez et al., 2022). The use of phenicols for tularemia meningitis seems no longer justified due to the risk of fatal aplastic anemia. These antibiotics might be useful in case of brain involvement.

#### 6.4.4 Endocarditis and aortic infections

*Francisella tularensis* endocarditis is a rare complication of tularemia and mostly occurred in men older than 55 years (Tancik and Dillaha, 2000; Salit et al., 2013; Yeom et al., 2015; Gaci et al., 2017; Olivo et al., 2019; Darmon-Curti et al., 2020; Kaeppler et al., 2020). *F. tularensis* infection involved native cardiac valves (Tancik and Dillaha, 2000; Salit et al., 2013; Gaci et al., 2017), a prosthetic valve (Kaeppler et al., 2020), or a pacemaker (Gaci et al., 2017). Beta-lactams (amoxicillin-clavulanate, piperacillin-tazobactam, cefotetan, cefazolin, ceftriaxone), erythromycin, and vancomycin were ineffective, including in some patients receiving gentamicin concomitantly (Tancik and Dillaha, 2000; Salit et al., 2013; Gaci et al., 2017; Kaeppler et al., 2020). Thus, the first-line treatments for endocarditis caused by *Staphylococcus* or *Streptococcus* species are not suitable for tularemia endocarditis. Most patients with tularemia endocarditis received gentamicin intravenously for at least 2 weeks. Ciprofloxacin or another fluoroquinolone was often combined to gentamicin or administered orally as a relay. The total duration of the specific treatment varied from 6 weeks to up to 12 weeks (Olivo et al., 2019). In some patients complications occurred while on appropriate antibiotic therapy, including a pulmonary embolism despite 14 days of intravenous gentamicin and ciprofloxacin (Olivo et al., 2019). Interestingly, in many patients, cardiac valve vegetations were still detected by echocardiography up to 6 months after the end of treatment (Salit et al., 2013; Olivo et al., 2019; Kaeppler et al., 2020). In one patient with severe complications (e.g., pulmonary embolism) and poor compliance to the antibiotic therapy, mitral valve replacement was performed. In other patients, persistence of cardiac vegetations was not associated with relapse and patients recovered without complications (Salit et al., 2013; Kaeppler et al., 2020). Although no definite recommendation can be raised from these anecdotal tularemia endocarditis cases, a 2-week course of intravenous gentamicin alone or combined with intravenous fluoroquinolone followed by 4 weeks oral administration of a fluoroquinolone seems a good alternative in the absence of severe complications. Cardiac surgery was rarely performed and mainly justified by a high risk of hemodynamic failure. Most patients were infected with *F. tularensis* type B and no death was reported.

Two cases of *F. tularensis* aortitis have been reported (Briere et al., 2016; Darmon-Curti et al., 2020; Kuzmova et al., 2023). A 85-year-old man developed fever and a systolic murmur due to minimal aortic insufficiency (Briere et al., 2016). Enhanced CT and FDG-PET/CT scans revealed an inflammatory infrarenal aortic aneurysm. After 5 days of vancomycin plus gentamicin, doxycycline was administered due to *F. tularensis* positive blood cultures. Nineteen days later, part of the aorta was replaced by an aorto-aortic allograft. *F. tularensis* was PCR detected in the removed aortic tissues. The patient further received 3 months of doxycycline plus levofloxacin and was considered cured. An aortic endograft infection was reported in a 79-year-old man (Kuzmova et al., 2023). *F. tularensis* was isolated from the aortic and periaortic tissues. He was cured after replacing the aortic graft and gentamicin plus ciprofloxacin treatment for 2 weeks then ciprofloxacin for 10 weeks

#### 6.4.5 Prosthetic joint infections

Clary et al. (2022) reported a finger interphalangeal joint arthritis caused by *Francisella tularensis* inoculated through a cat bite. The patient was cured after receiving gentamicin for 4 weeks. Most reported osteoarticular infections involved knee or hip prostheses, in persons aged 49–84 years, and more frequently men than women. These infections occurred from 1 month up to 25 years after surgical placement of the prosthesis (Cooper et al., 1999; Rawal et al., 2017; Chrdle et al., 2019; Azua and Voss, 2020; Darmon-Curti et al., 2020; Ponderand et al., 2023). Suspected modes of infection included cleaning a rabbit barn (Chrdle et al., 2019), oral contamination (abdominal symptoms), gardening (Chrdle et al., 2019), respiratory infection (Ponderand et al., 2023), skin abrasion after falling (Darmon-Curti et al., 2020), and tick bite (Cooper et al., 1999). Symptoms mainly included fever, joint pain, and difficulty in moving the infected joint. Diagnosis was often delayed of several weeks to months (Cooper et al., 1999) and usually established by *F. tularensis* isolation from osteoarticular or blood samples. Local complications were occasionally observed [e.g., hip hematoma (Darmon-Curti et al., 2020)], but no systemic complication which might be related to the fact that only *F. tularensis* type B strains were involved. Cure was usually obtained by combining an antibiotic treatment with surgical replacement of the infected joint prosthesis. Four patients were reported to be cured after doxycycline treatment (Rawal et al., 2017; Chrdle et al., 2019; Azua and Voss, 2020; Escovar et al., 2023). One patient was cured after receiving doxycycline for 6 weeks plus knee prosthesis replacement (Chrdle et al., 2019). Another patient denied surgery and was considered cured only after receiving doxycycline for 1 year (Rawal et al., 2017). The last patient also received a long term treatment with doxycycline (Azua and Voss, 2020). A patient with infected knee prosthesis was administered a lifelong doxycycline treatment because the prosthesis could not be totally removed (Ponderand et al., 2023). A patient with knee prosthesis infection who denied surgical treatment relapsed after receiving 10 days of gentamicin and 21 days of doxycycline, but then was cured with ciprofloxacin for 20 days (Chrdle et al., 2019). Some patients received an antibiotic therapy (e.g., doxycycline for 3 months, gentamicin, or ofloxacin for 6 weeks), then the infected prosthesis was replaced and antibiotics were further administered after surgery (e.g., ciprofloxacin for 3 months, ciprofloxacin plus doxycycline for 9 weeks, or amikacin for 5 days plus ciprofloxacin for 6 weeks) (Darmon-Curti et al., 2020; Ponderand et al., 2023). A complex case was reported in a 68-year-old man with a knee arthroplasty because of rheumatic arthritis (Cooper et al., 1999). The patient first experienced a postoperative prosthesis infection caused by *Enterococcus faecalis*. One year later, knee inflammation led to prosthesis removal with insertion of a joint spacer with erythromycin-containing cement. After several antibiotic courses with poor clinical response his symptoms improved under ciprofloxacin plus rifampicin combination. A new total knee arthroplasty was placed 3 months later. Diagnosis of tularemia was available at that time likely after several months of evolution of the disease. The patient further received 6 months of ciprofloxacin plus rifampicin and was considered cured. Overall, the therapeutic management of *F. tularensis* prosthetic joint infections was highly variable between patients, reflecting diagnostic difficulties and the need for treatment adaptation to the context and clinical evolution of each patient. However, all patients were considered cured from their *F. tularensis* infection.

#### 6.4.6 Others

Many other complications can occur in tularemia patients, including skin necrosis (Syrjälä et al., 1984), tonsillar phlegmon and otitis (Gürkov et al., 2009; Guerpillon et al., 2016), myocarditis and pericarditis (Landais et al., 2008; Frischknecht et al., 2019; Darmon-Curti et al., 2020), and pleurisy and pulmonary abscess (Gill and Cunha, 1997; Thomas and Schaffner, 2010). The cases reported for each type of complication are too few to deduce a therapeutic approach.

### 6.5 Tularemia in children

Tularemia occurs less frequently in children than in adults, mainly due to a lower risk of exposure to infection sources (e.g., animal contacts and tick bites). Children are more likely to be infected when the predominant mode of infection is mosquito bite or consumption of contaminated water (Eliasson and Bäck, 2007; Kaya et al., 2011; Gozel et al., 2014; Tezer et al., 2015; Karlı et al., 2018; Dryselius et al., 2019). Data on tularemia treatment in children are summarized in Table 9.

**TABLE 9:** Antibiotic efficacy in children with tularemia.

References	Country	Patients’ number, male/ female, age range or mean	Number or % of clinical forms	Antibiotics administered (number or % of patients)	Lymph node suppuration and surgery (number, % of patients)	Failures, relapses, and complications (number, % of patients)
Gozel et al., 2014	Turkey	26 children (15m/11f, 3–16y)	OP	str (11), cip (11), dox (1), str + dox (3).	Drainage (8/26, 30.7%)	
Kaya et al., 2011	Turkey	11 children (7m/4f, 8–17y)	OP	Gen (12–18 days), then str (14 days) in 4 cases with relapse	Drainage in 4 cases with relapse	Failure (4/11, 36.4%); No improvement after 7–10d gen and ceftazidime (7/11, 63.6%) but cure after str 14d
Centers for Disease Control and Prevention (CDC), 2009	USA	73 children	UG (19), GL (20), OG (3), OP (1), PN (2)	Known for 47. Amg (29), flq (18), tet/dox (8), often combined	Surgery (9/49, 18.4%)	4 relapses (49 known outcome, 8.2%), multiple complications in one case
Cross et al., 1995	USA	23 children (16m/7f, 16mths-16y)	Most UG and GL after tick bite	Str, gen, tetra, alone or combined		No relapse
Johansson et al., 2000	Sweden	12 children (1–10y)	UG	Cip, 14–14d		
Tezer et al., 2015	Turkey	100 children, 63m/37w, mean age, 10.1y (3–18y)	90 OP, 7 UG, 3 OG	gen (56), str (1), dox (23), cip (20), 10–14d	Suppuration (9, 9%); surgery (43, 43%)	Relapses (26, 26%)
Karlı et al., 2018	Turkey	19 children, 3.5–17y (mean, 10.5y)	100% OP with antibiotic treatment failure	str (9), cip (5), gen (4), dox (1), then cip 4 weeks for all	Aspiration (2), drainage (9), excision (5), overall (16/19, 84.2%)	Cases with treatment failure after str, cip, gen, or dox

str: streptomycin, gen: gentamicin, amg: aminoglycoside, tet: tetracycline, dox: doxycycline, flq: fluoroquinolone, cip: ciprofloxacin. Tularemia forms: ulceroglandular (UG), glandular (GL), oropharyngeal (OP), oculoglandular (OG), pneumonic (PN), and typhoidal (TY).

A few case series of pediatric tularemia have been reported [Cross et al., 1995; Johansson et al., 2001; Centers for Disease Control and Prevention (CDC), 2009; Kaya et al., 2011; Gozel et al., 2014; Tezer et al., 2015; Karlı et al., 2018]. The above case series included 264 tularemia cases in children aged 16 months to 18 years, with a male predominance (60.2% of 160 patients with known gender). The clinical form was known for 230 (87.1%) cases, including 63.5% OP and 36.5% UG and GL forms, reflecting more frequent infection via the consumption of contaminated water (Kaya et al., 2011; Gozel et al., 2014; Tezer et al., 2015) or mosquito bites (Eliasson and Bäck, 2007; Dryselius et al., 2019). Many children received inappropriate empirical antibiotic therapy with poor clinical response, including beta-lactams (amoxicillin, amoxicillin-clavulanate, piperacillin-tazobactam, cefaclor, cefuroxime, cefotaxime, ceftriaxone) and macrolides (erythromycin, azithromycin, clarithromycin) (Garver et al., 1994; Cross et al., 1995; Arav-Boger, 2000; Kaya et al., 2011; Sobolewska-Pilarczyk et al., 2014; Tezer et al., 2015; Passiouk and Heininger, 2016; Nemmour et al., 2019). Effective antibiotics administered (known for 180 cases, 68.2%) included an aminoglycoside (gentamicin or streptomycin) in 60.6% of cases, a tetracycline in 18.3%, and a fluoroquinolone in 27.2%, often administered combined or sequentially. It should be noted that doxycycline and fluoroquinolones are no longer formally contraindicated and are generally well tolerated in young children (before 8 years) for short-term treatments. Treatment with intravenous aminoglycoside (streptomycin or gentamicin for 10–14 days) was often followed by oral ciprofloxacin or doxycycline (for 2–3 weeks) (Kosker et al., 2013; Formińska et al., 2015; Nemmour et al., 2019; Kocabaş et al., 2020). In many cases, ciprofloxacin alone was effective (Arav-Boger, 2000; Johansson et al., 2000; Passiouk and Heininger, 2016; Wetzstein et al., 2019). Treatment failures or relapses were reported in 34 (12.8%) and lymph node surgery in 80 (30.3%) of the 264 cases. Occasionally, cure was only obtained after several weeks to months of antibiotic treatment combined with suppurated lymph node surgery (Kosker et al., 2013; Nemmour et al., 2019; Kocabaş et al., 2020). Karlı et al. (2018) reported 19 pediatric OP tularemia cases that had not responded to the antibiotic treatments administered. Treatment failures corresponded to enlargement of lymph nodes or development of new ones, lymph node suppuration, persistent or recurrent fever, or persistent elevated inflammatory markers. Most of these patients required lymph node surgery for cure. Although pediatric tularemia cases had variable severity and evolution length, no death was reported among these 264 pediatric tularemia cases.

Sporadic cases of pediatric tularemia have reported similar findings but some unusual presentations warrant further discussion (Garver et al., 1994; Kosker et al., 2013; Sobolewska-Pilarczyk et al., 2014; Nemmour et al., 2019; Wetzstein et al., 2019; Kocabaş et al., 2020). An 8-year-old boy with fever and granulomatous hepatitis was only cured after receiving 14 days of streptomycin (14d) plus 21 days of ciprofloxacin, then 10 days of gentamicin and 1 month of ciprofloxacin (Kocabaş et al., 2020). A 5-year-old boy with OP tularemia dramatically improved after 10 days of intravenous gentamicin (6 mg/kg/day) (Nemmour et al., 2019). However, he relapsed 2 weeks later with fever, dysphagia and worsening of neck swelling. An MRI exam revealed a left parapharyngeal abscess and necrotic cervical lymph nodes. He was cured after drainage of the parapharyngeal abscess and resection of suppurated lymph nodes and an antibiotic therapy with intravenous gentamicin (6 mg/kg/day, 2 weeks) followed by oral doxycycline for 2 weeks. A 15-year-old boy developed OG tularemia with submandibular and preauricular lymphadenopathy, and subsequent infection of the parotid gland and masseter muscle (Kosker et al., 2013). Lymph node swelling persisted despite 14 days of gentamicin. He then received streptomycin plus ciprofloxacin rapidly replaced by tetracycline due to fluoroquinolone allergy. Cure was obtained by combining lymph node excision, and streptomycin for 10 days plus tetracycline for 8 weeks. A 5-year-old boy developed UG tularemia with abdominal pain 2 weeks after tick bites (Garver et al., 1994). He received gentamicin (7.5 mg/kg/day) and oxacillin (200 mg/kg/day). However, radiological exams revealed bilateral peri-hilar infiltrates, cervical lymphadenopathy, spleen nodules, and mild hepatomegaly. He recovered after excision of suppurated lymph nodes and gentamicin for 7 days. Interestingly, splenic nodules persisted for several months. A 11-year-old Polish boy with UG and PN tularemia received empirical treatment with cefotaxime, clarithromycin, and clindamycin for 11 days without improvement (Sobolewska-Pilarczyk et al., 2014). He was cured after receiving gentamicin (10 mg/kg/day) plus amoxicillin/clavulanate (3.6 g/day, IV, 7 days), followed by ceftriaxone (2 g/d, 6 days) plus cotrimoxazole (1,920 mg/d, 10 days).

### 6.6 Tularemia in pregnant women

Tularemia cases occurring in pregnant women remain scarce (Table 10). Two UG tularemia cases occurring in pregnant women after contact with rabbits during their 16th week of gestation were reported in the USA before the antibiotic era (Bricker, 1931; Bowe and Wakeman, 1936). One experienced spontaneous abortion and the second premature birth. More recently, Ata et al. (2013) reported an OP tularemia case in a 36-year-old women at 6 weeks of gestation. Empirical treatment with amoxicillin-clavulanate for 10 days was ineffective. She denied any specific antibiotic therapy and pregnancy evolved to intrauterine fetal death. These cases highlight the potential obstetrical complications of untreated tularemia in pregnant women.

**TABLE 10:** Antibiotic efficacy in pregnant women with tularemia.

References	Country	Patient’s age (years) and weeks of gestation	Number or % of clinical forms	Antibiotics administered (number or % of patients)	Surgery for lymph node suppuration
Charles et al., 2008	USA	Pregnant, 29y	UG, tick-borne	Dox (1wk), then amc, azt, and dox (2 weeks). Became pregnant. Josamycin po, 3 weeks	Several needle aspirations
Dentan et al., 2013	France	Pregnant, 27y, 6 weeks	OP, skinning hunted rabbits	Amx for 3 weeks. Then, azt for 6 weeks	Lymph node excision
Yeşilyurt et al., 2013	Turkey	Pregnant, 26y, 18 weeks	OP	Ceftriaxone, then gen 10d, then cip 2 weeks	Several lymph node drainages
Yeşilyurt et al., 2013	Turkey	Pregnant, 31y, 23 weeks	OP+OG	2nd generation cephalosporin for 7d, then gen 10d, then cip 10d	Several lymph node drainages
Yeşilyurt et al., 2013	Turkey	Pregnant, 29y, 27 weeks	OP	Amc 7d, then gen, then cip 2 weeks	Lymph node drainage
Yeşilyurt et al., 2013	Turkey	Pregnant, 35y, 30 weeks	OP	Amc 10d, then gen, then cip 2 weeks	Lymph node drainage
Celik et al., 2013, 2014	Turkey	Pregnant, 18y, 16 weeks	OG	Cefuroxime 6 weeks	Lymph node drainage
Celik et al., 2013, 2014	Turkey	Pregnant, 27y, 18 weeks	OG	Amc, and eye drops (gen+cip)	Dacryocystitis and lymph node drainage
Johnsrud et al., 2019	USA	Pregnant, 26y, 18 weeks	UG, cat bite	Amx, azt	Lymph node drainage
Saranovic et al., 2023	Serbia	Pregnant, 33y, 18 weeks	GL	Gen 7d	Lymph node excision

All cases had good pregnancy outcome and gave birth to a healthy infant. Amx: amoxicillin; amc: amoxicillin plus clavulanic acid; azt: azithromycin; gen: gentamicin, dox: doxycycline, cip: ciprofloxacin. Tularemia forms: ulceroglandular (UG), glandular (GL), oropharyngeal (OP), oculoglandular (OG), pneumonic (PN), and typhoidal (TY).

In contrast, most of recent *F. tularensis* infections in pregnant women had good pregnancy outcome with healthy infants (Charles et al., 2008; Celik et al., 2013, 2014; Dentan et al., 2013; Yeşilyurt et al., 2013; Johnsrud et al., 2019; Saranovic et al., 2023). Tularemia cases corresponding to the above references involved ten pregnant women, aged 18–35 years (mean, 28.1 years), at 6–30 weeks of gestation (mean, 19.5 weeks), suffering from OP (4 cases), OP plus OG (1), OG (2), GL (1), or UG (2) forms. Beta-lactams were often administered empirically with poor efficacy, including amoxicillin, amoxicillin-clavulanate, cefuroxime, and ceftriaxone (Charles et al., 2008; Celik et al., 2013, 2014; Dentan et al., 2013; Yeşilyurt et al., 2013; Johnsrud et al., 2019). Doxycycline was not administered during pregnancy due to potential risk of tooth (discoloration of teeth and enamel hypoplasia) and bone toxicity to the fetus. Four patients with OP form of tularemia were treated with gentamicin for 10 days followed by ciprofloxacin for 2 weeks (Yeşilyurt et al., 2013). One patient with a GL form received 7 days of gentamicin (Saranovic et al., 2023).

Although gentamicin can induce congenital deafness, and ciprofloxacin and other fluoroquinolones have been associated with teratogenic effects in animal models (Johnsrud et al., 2019), none of the five mothers receiving these antibiotics nor their infants had side effects (Yeşilyurt et al., 2013; Saranovic et al., 2023). Gentamicin remains the first-line treatment in pregnant women with severe tularemia (Dennis et al., 2001; Bossi et al., 2004). Azithromycin was administered during pregnancy in two patients with OP or UG forms, respectively (Dentan et al., 2013; Johnsrud et al., 2019) and josamycin in one patient with an UG form (Charles et al., 2008). These macrolides are considered moderately active against *F. tularensis*, but they likely represent a safe alternative in pregnant women with mild forms of tularemia in areas where *F. tularensis* type B bv II strains are absent.

All pregnant women developed suppurated lymph nodes requiring (often several) needle aspirations, drainages, or excision. One patient also required dacryocystitis drainage (Celik et al., 2013). Overall, none of the pregnant patients treated with antibiotics and surgical management of suppurated lymph nodes had obstetrical complications and their infants were healthy (Charles et al., 2008; Celik et al., 2013, 2014; Dentan et al., 2013; Johnsrud et al., 2019; Saranovic et al., 2023).

### 6.7 Tularemia in the immunocompromised

Tularemia has occasionally been reported in patients with an immunosuppressive disease or treatment (Limaye and Hooper, 1999; Naughton et al., 1999; Sarria et al., 2003; Khoury et al., 2005; Mittalhenkle and Norman, 2006; Faucon et al., 2011; Ozkok et al., 2012; Weile et al., 2013; Calin et al., 2017; Bahuaud et al., 2019; Table 11). The above cases occurred in the USA, France, Germany, and Turkey, in patients of 24–69 years (mean age, 49.8 years), with a male predominance (8 of 9 cases for which gender was specified). Most patients had received a transplant, either kidney (4 cases), liver (2), heart (1), peripheral blood (2) or bone marrow stem cells (1) (Limaye and Hooper, 1999; Naughton et al., 1999; Sarria et al., 2003; Khoury et al., 2005; Mittalhenkle and Norman, 2006; Faucon et al., 2011; Ozkok et al., 2012; Weile et al., 2013; Bahuaud et al., 2019). Most of these patients were under immunosuppressive treatment at the time of infection, including various combinations of prednisone, prednisolone, mycophenolate mofetil, cyclosporin A, tacrolimus, rapamycin. One patient with severe psoriatic arthritis had received methotrexate and certolizumab for 5 years before infection (Calin et al., 2017). Another patient had AIDS and chronic active hepatitis C (Limaye and Hooper, 1999). *F. tularensis* infection occurred between 2 days (Sarria et al., 2003) up to 15 years (Faucon et al., 2011) post-transplantation. Most infections were pneumonic forms of tularemia (9 patients) but often with extrapulmonary symptoms suggesting systemic dissemination of *F. tularensis* to the lungs and other organs whatever the portal of entry of bacteria. One patient developed, 2 days after allogenic bone marrow transplantation for acute lymphoblastic leukemia, fever, lethargy, severe neutropenia, inguinal lymphadenopathy, and evolved to coma and acute renal failure and died (Sarria et al., 2003). Another patient developed glandular tularemia with fever and right elbow swelling (Calin et al., 2017). Overall, tularemia diagnosis was established by the isolation of *F. tularensis* from blood samples, lymph nodes, a lung nodule, or an elbow joint fluid (Limaye and Hooper, 1999; Naughton et al., 1999; Sarria et al., 2003; Ozkok et al., 2012; Weile et al., 2013; Calin et al., 2017; Bahuaud et al., 2019). Frequent isolation of *F. tularensis* from infected immunocompromised patients is in sharp contract with the 10% rate isolation observed in immunocompetent patients (Maurin and Gyuranecz, 2016), suggesting more frequent systemic infections in the former due to lower control of bacterial multiplication.

**TABLE 11:** Antibiotic efficacy in immunocompromised patients with tularemia.

References	Country	Patients	Clinical form	Antibiotic treatment and outcome
Limaye and Hooper, 1999	USA	Man, 50 years, liver transplant (hepatitis C and alcohol-related cirrhosis)	Pneumonic, 3 years post-transplantation, while under prednisone and azathioprine	Cured with levofloxacin, 21 days
Limaye and Hooper, 1999	USA	Man, 33 years, AIDS and chronic active hepatitis C	Pneumonic	Cured with levofloxacin, 15 days
Naughton et al., 1999	USA	Syngeneic peripheral blood stem cell transplant	Pneumonic, 7 months after transplantation	Cured with ciprofloxacin
Sarria et al., 2003	USA	Man 43 years, allogenic bone marrow transplantation	Systemic infection, 2 days after transplantation	Imipenem plus vancomycin, then gentamicin added, coma and acute renal failure, death
Weile et al., 2013	Germany	Man 54 years, stem cell transplant for acute myeloid leukemia	Pneumonic, 4 years post-transplantation, while under tacrolimus, steroids, and levofloxacin prophylaxis (125 mg/d) due to chronic graft-versus-host-disease	Cured after imipenem (IV, 1,500 mg/d) and levofloxacin (IV, 500 mg/d), then doxycycline per os
Calin et al., 2017	France	Woman, 32 years, certolizumab and methotrexate for 5 years due to severe psoriatic arthritis	Fever and right elbow swelling 11 weeks after contact with rabbits	Gentamicin 14 days plus ciprofloxacin 28 days, but recurrence 2 days after treatment withdrawal, new axillary lymph nodes, cure after 4 months of ciprofloxacin plus doxycycline
Bahuaud et al., 2019	France	51 years, heart transplant	Pneumonic, while under prednisolone (1 mg/day), cyclosporin (80 mg bid), and mycophenolate mofetil (1,500 mg bid).	Ceftriaxone plus metronidazole, then pyrimethamine/ sulfadiazine, then cotrimoxazole. Recurrence 4 months later. Cured after mediastinal lymph node resection and ciprofloxacin (750 mg bid) plus gentamicin (300 mg) for 7 days, then ciprofloxacin for 14 days
Bahuaud et al., 2019	France	64 years, liver transplant for alcoholic and viral cirrhosis	Pneumonic, acute respiratory distress and septic shock while under tacrolimus (0.5 mg) and mycophenolate mofetil (500 mg tid)	Improved by ceftriaxone, spiramycin, and gentamicin (2 doses). Cured after ciprofloxacin (500 mg bid) for 14 days
Ozkok et al., 2012	Turkey	Man, 24 years, kidney transplantation	Glandular, 1 year after transplantation	Cured after doxycycline (100 mg twice daily) for 4 weeks
Khoury et al., 2005	USA	Man, 69 years, kidney transplant for renal failure due to polycystic kidney	Pneumonic plus abdominal symptoms, 4 years post transplantation, while under prednisone, mycophenolate mofetil, rapamycin	Cured after doxycycline for 14 days
Mittalhenkle and Norman, 2006	USA	59 years, kidney transplant due to polycystic kidney	Pneumonic, 11 years post-transplantation, while under prednisone, mycophenolate mofetil, and cyclosporine A	Clinical improvement after fluoroquinolone
Faucon et al., 2011	France	Man, 69 years, kidney transplant for IgA nephropathy	Pneumonic, 15 years post-transplantation, while under prednisolone, mycophenolate mofetil, cyclosporine A	Cured after levofloxacin (500 mg/day) for 14 days

Empirical treatments with ceftriaxone, imipenem, metronidazole, pyrimethamine-sulfadiazine, cotrimoxazole, vancomycin, and spiramycin were ineffective (Sarria et al., 2003; Bahuaud et al., 2019). Cure was usually obtained after 2–4 weeks of gentamicin, doxycycline, ciprofloxacin, levofloxacin, or combinations of these antibiotics (Limaye and Hooper, 1999; Naughton et al., 1999; Khoury et al., 2005; Mittalhenkle and Norman, 2006; Faucon et al., 2011; Ozkok et al., 2012; Weile et al., 2013; Calin et al., 2017; Bahuaud et al., 2019). One patient required resection of suppurated mediastinal lymph nodes for cure (Bahuaud et al., 2019). Another patient was cured after drainage of infected elbow joint fluid and 4 months treatment with ciprofloxacin plus doxycycline combination (Calin et al., 2017). Only one patient died (Sarria et al., 2003).

## 7 Discussion

The aim of this review was to summarize current literature on treatment of human tularemia and highlight the inadequacy of current recommendations, the primary objective of which is the management of multiple and often serious infections in a context of biological threat (Dennis et al., 2001; Bossi et al., 2004). Human infections with *F. tularensis* greatly vary in their sources and modes of infection, clinical presentation, diagnosis and treatment delays, evolution, and outcome, and according to the patients’ underlying health and immune status before infection.

There are copious data available to confirm that the beta-lactams, most macrolides, lincosamides, synergistins, and first-line antituberculosis drugs (except streptomycin) are almost ineffective in tularemia patients (Arikan et al., 2003; Sencan et al., 2009; Willke et al., 2009; Cağlı et al., 2011; Mahy et al., 2011; Maurin et al., 2011; Altuntas et al., 2012; Jackson et al., 2012; Erdem et al., 2014; Sobolewska-Pilarczyk et al., 2014; Boone et al., 2015; Formińska et al., 2015; Pekova et al., 2017; Whitten et al., 2017; Darmon-Curti et al., 2020; Haulrig et al., 2020; Kubiliute et al., 2021). Failure of treatment with beta-lactams may even suggest a diagnosis of tularemia in a compatible clinical and epidemiological context. Tularemia patients with chronic lymphadenopathy (especially in the mediastinal and hilar areas) were occasionally misdiagnosed as tuberculosis cases but failed to respond to antituberculosis therapy.

The aminoglycosides remain the first line antibiotics advocated to treat severe tularemia cases, including severe pneumonic and typhoidal forms, especially when caused by the most virulent type A strains (Dennis et al., 2001; Bossi et al., 2004). Streptomycin and gentamicin have likely equivalent effectiveness in patients with systemic diseases. Streptomycin is no longer available in many countries, and usually not administered to young children and pregnant women because of higher nephrotoxicity and ototoxicity compared to gentamicin. Some other aminoglycosides [e.g., tobramycin (Heine et al., 2017; Caspar et al., 2018)] have similar *in vitro* activity against *F. tularensis*, but experience in treating tularemia with these molecules is too scarce to consider them as alternatives. Since end of the 1990’s aminoglycoside are administered once daily rather than in multiple dosing regimen. Single dose administration evidenced similar efficacy along with reduced probability of nephrotoxicity (Eliopoulos et al., 2007; Avent et al., 2011). The WHO guidelines still recommend administration of gentamicin in two doses while one daily dose in recommended in the consensus statement from Dennis et al. (2001).

Fluoroquinolones or tetracyclines are considered first line antibiotics in patients with mild to moderate severity diseases (Tärnvik and Chu, 2007; Hepburn and Simpson, 2008; Maurin, 2020; Wawszczak et al., 2022). Most of these patients suffer from local or regional infection with lymphadenopathy. The outstanding question is whether one of these two classes of antibiotics is more effective and should be considered as a priority in the treatment of these glandular forms of tularemia. Ciprofloxacin is by far the most prescribed fluoroquinolone, while levofloxacin and moxifloxacin are also considered effective. In recent years, doxycycline was almost the only tetracycline administered to tularemia patients. Arguments that may be in favor of prescribing a fluoroquinolone rather than doxycycline are: (1) fluoroquinolones are bactericidal against *F. tularensis in vitro* while doxycycline is only bacteriostatic (Maurin et al., 2000; Caspar and Maurin, 2017); (2) fluoroquinolones are more effective in animal models than doxycycline, especially for severe infections caused by type A strains (Russell et al., 1998; Rotem et al., 2012); and (3) lower relapse and failure rates are usually reported in humans treated with fluoroquinolones compared to tetracyclines (Pérez-Castrillón et al., 2001; Meric et al., 2008; Erdem et al., 2014). However, some studies have reported fewer relapses with doxycycline compared to fluoroquinolones (Darmon-Curti et al., 2020). In many patients, treatment failures and relapses are related to the site of infection (e.g., prosthetic infection), treatment delay higher than 2–3 weeks, and the occurrence of complications such as lymph node suppuration (Meric et al., 2008; Komitova et al., 2010), rather than the antibiotic treatment administered. In many reports, success rates were higher in patients treated within the first 3 weeks following disease onset (Helvaci et al., 2000; Celebi et al., 2006; Komitova et al., 2010; Gönen, 2013; Tezer et al., 2013; Ulu-Kilic et al., 2013; Oz et al., 2014). In a study in Bursa (Turkey), success rate whatever the antibiotic regimen chosen was 22/31 (66%) before 3 weeks and but only 21/67 (31%) after 3 weeks. Similarly, Meric et al. (2008) showed by logic regression analyses of 145 cases, that instauration of treatment after the 2nd week doubled therapeutic failure rate. Indeed, complications such as persistence of lymphadenopathies, abscess formation, suppuration or necrosis are fewer if treatment is started during the first 3 weeks (Helvaci et al., 2000; Eliasson and Bäck, 2007; Chitadze et al., 2009; Oz et al., 2014). In particular, Oz et al. (2014) observed 38% abscess formation in 55 children when treatment began early, increasing to 62% after a 3-week delay. In Konya (Turkey), treatment of 16 oropharyngeal tularemia was achieved by antibiotic therapy alone in 9 cases diagnosed within 3 weeks whereas the 7 remaining cases for which delay was superior to 3 weeks, all required lymph node excision (Tezer et al., 2013).

Hence, treatment failures and relapse rates are often high in case series, but highly depends on the included patients (Evans et al., 1985; Meric et al., 2008; Maurin et al., 2011; Weber et al., 2012; Ulu-Kilic et al., 2013; Erdem et al., 2014; Darmon-Curti et al., 2020). Many other criteria may determine the choice of a fluoroquinolone or a tetracycline, in particular the history of allergy or intolerance to these drugs, possible side effects, the patient’s medical status, age, etc.

It should be emphasized that in many patients with lymph node suppuration or other complications, several antibiotic courses were administered using different antibiotic classes, in combination or alternatively. This practice was either systematic or dictated by an unfavorable and prolonged evolution of the infection. In this case, it is likely that the effectiveness of the treatment corresponded to the cumulative effect of the different antibiotic courses, and ultimately to the total duration of antibiotic therapy. In any case, it is difficult to compare the effectiveness of successive antibiotic therapies in this context, especially since a surgical treatment (e.g., for resection of suppurated lymph nodes) was often added to the medical care. It should be stressed that acquired resistance to antibiotics has never been demonstrated in *F. tularensis* which currently allows several courses with the same antibiotic in each patient.

Specific situations include tularemia occurring in children, pregnant women, and immunocompromised patients. Gentamicin is still advocated as first line treatment for severe systemic infections. In children, a fluoroquinolone or doxycycline can be administered. Although side effects are rare, strict medical surveillance is mandatory. Fluoroquinolones and tetracyclines are classically contraindicated in pregnant women, although fluoroquinolones have been used successfully and without severe side effects in a few pregnant women with tularemia (Yeşilyurt et al., 2013). Azithromycin or josamycin, occasionally combined with drainage or resection of suppurated lymph nodes has been reported as successful in a few pregnant women in areas where type B biovar II strains are absent (Charles et al., 2008; Dentan et al., 2013; Johnsrud et al., 2019). Further evaluation of such antibiotic treatment is needed. Tularemia in immunocompromised patients is often a systemic life-threatening disease, which needs to be systematically discussed in endemic areas. Treatment in immunocompromised hosts is like that in immunocompetent patients, but a longer duration of antibiotic administration depending on treatment delay should likely be considered.

Almost every organ can be involved in patients experiencing *F. tularensis* bacteremia. The antibiotic treatment is still based on the aminoglycosides (streptomycin or gentamicin), the fluoroquinolones, and doxycycline. However, because these complications are rare, only anecdotal reports are available. For each complication, the optimum antibiotic treatment remains to be established, especially the antibiotic or antibiotic combination to be used, and the treatment duration. Other antibiotic classes have been considered for treatment of these complications. Chloramphenicol has been used with success in patients with tularemia meningitis (Hofinger et al., 2009). Because of the risk of fatal aplastic anemia, the prescription of this antibiotic must be limited, and its usefulness well assessed. The use of rifampicin in combination with another first-line antibiotic has been reported, especially for osteoarticular infections (Ponderand et al., 2023). None of the other available antibiotic classes have been reported as useful for treating tularemia.

A significant number of patients with complicated forms of tularemia require surgical management of suppurated lymphadenopathy to achieve cure (Cağlı et al., 2011; Maurin et al., 2011; Kızıl et al., 2012; Weber et al., 2012; Karakas et al., 2014; Rimawi et al., 2014; Fachinger et al., 2015; Darmon-Curti et al., 2020). Most often, this surgery is considered after several antibiotic treatments have failed. Although needle aspiration of lymph node suppuration can be proposed, resection of suppurated lymph nodes is usually more effective (Kızıl et al., 2012). It is likely that earlier surgical management of these patients would shorten evolution and thus the highly disabling nature of these infections. The development of criteria for surgical intervention could help in this process. Surgery is also needed for many other rare situations, including skin and soft tissue infections, osteoarticular infections, tonsillar phlegmon, periorbital infection, endocarditis, aortitis, among others (Cooper et al., 1999; Centers for Disease Control and Prevention (CDC), 2009; Gürkov et al., 2009; Thomas and Schaffner, 2010; Junkins and Snyder, 2011; Rimawi et al., 2014; Fachinger et al., 2015; Arslan et al., 2016; Briere et al., 2016; Gaci et al., 2017; Azua and Voss, 2020; Darmon-Curti et al., 2020, 2020; Kaeppler et al., 2020; Ponderand et al., 2023).

Due to lack of information and insufficient comparable data, no meta-analyses can be performed to date to draw strong conclusions on therapeutic failure rate by clinical form, antibiotic treatment administered, or treatment delay. To reach this goal, studies should at least give the following data for each reported case independently to be able to aggregate all the data: age, sex, clinical form of tularemia, antibiotic treatment (molecules, doses, duration of administration), appropriate treatment delay after disease onset, lymph node suppuration with spontaneous or induced drainage, surgical procedures, and disease evolution (therapeutic failure or relapse, death). Therefore, therapeutic failure and relapse should be clearly defined. Although this review does not make it possible to construct solid therapeutic recommendations, we have tried to establish proposals considering current literature data (Table 12). These proposals must be discussed by a group of experts to develop new recommendations adapted to the diversity of clinical situations encountered during tularemia.

**TABLE 12:** Antibiotic treatment suggestions for treating tularemia (doses are for adults) according to clinical form based on current literature.

Clinical form	Antibiotics	Dose and duration*	Additional treatment	Comments
Acute and severe infection, *F. tularensis* bacteremia, especially if type A (PN, TY, others)	1st line: gentamicin ($)	3 to 8 mg/kg once daily, i.v., 7–10 days	Symptomatic treatment. Intensive care admission if needed	Ciprofloxacin can be used in combination with gentamicin or as a relay of gentamicin
	2nd line: ciprofloxacin (or levofloxacin or moxifloxacin)	400 mg bid i.v. or 500 mg bid p.o. if possible		
UG, GL, OP	1st line: ciprofloxacin (or levofloxacin or moxifloxacin)	500 mg bid p.o., for 2–3 weeks or 3–4 weeks if treatment delay > 3 weeks after disease onset	Surgical resection or drainage, or fine-needle aspiration of suppurated lymph nodes Surgical treatment of associated skin and soft tissue infections, especially subcutaneous abscess, cellulitis, hand phlegmon, etc.	Surgery to be considered early after antibiotic treatment failure or relapse Higher treatment failure and relapse rates with doxycycline
	2nd line: doxycycline	200 mg once daily p.o., 3–4 weeks		
	3rd line: gentamicin ($)	4 to 7 mg/kg once daily, 7–10 days		
OG	1st line: ciprofloxacin (or levofloxacin or moxifloxacin)	500 mg bid p.o., for 2–3 weeks or 3–4 weeks if treatment delay > 3 weeks after disease onset	Local antibiotic therapy: ciprofloxacin or tobramycin drops for 7–10 days (1–2 drops per eye every 2 h for 2 days then every 4 h during daytime) Surgical treatment of suppurated lymphadenopathy or ocular complication (dacryocystitis, periorbital abscess, etc.)	Gentamicin combined with ciprofloxacin or doxycycline in case of complications (e.g., keratitis, dacryocystitis, and orbital cellulitis)
	2nd line: doxycycline	200 mg once daily p.o., 3–4 weeks		
	3rd line: gentamicin ($)	3 to 8 mg/kg once daily, i.v., 7–10 days		
PN (subacute or chronic)	1st line: ciprofloxacin (or levofloxacin or moxifloxacin)	400 mg bid i.v. or 500 mg bid p.o. if possible	Surgical treatment of suppurated mediastinal or hilar lymphadenopathy, lung abscess, pleural empyema, etc.)	Gentamicin combined with ciprofloxacin or doxycycline in case of complications (suppurated deep lymphadenopathy, lung abscess, pleurisy)
	2nd line: doxycycline	200 mg once daily p.o., 3–4 weeks		
	3rd line: gentamicin ($)	3 to 8 mg/kg once daily, i.v., 7–10 days		
Endocarditis	1st line: gentamicin plus ciprofloxacin	Usual dosages, i.v., 15 days for gentamicin and 4–6 weeks for ciprofloxacin (or more for prosthetic valve endocarditis or according to disease evolution)	Cardiac valve or prosthetic valve replacement if needed. Some patients did not relapse although the infected native or prosthetic cardiac valve was not removed.	Ciprofloxacin can be used in combination with gentamicin or as a relay of this antibiotic. Some patients were cured after only receiving ciprofloxacin
	2nd line: ciprofloxacin	500 mg bid, i.v. then p.o., 4–6 weeks		
Osteoarticular infection without prosthesis	1st line : ciprofloxacin	500 mg bid, p.o., 4–6 weeks	Surgical treatment if needed	Very few data available
	2nd line : doxycycline	200 mg p.o. once daily, 4–6 weeks		
Osteoarticular infection on prosthesis	1st line: gentamicin plus ciprofloxacin	Usual dosages, 7–10 days for gentamicin i.v. and 4–6 weeks for ciprofloxacin p.o., then prosthetic replacement and ciprofloxacin at least 6 weeks	Prosthesis replacement. The time of prosthesis replacement greatly varied between patients.	Few data available. Patients who denied prosthesis removal long-term doxycycline treatment was proposed
	2nd line: gentamicin plus doxycycline	Usual dosages, 7–10 days for gentamicin i.v. and 4–6 weeks for doxycycline p.o., then prosthetic replacement and doxycycline at least 12 weeks		
Meningitis, encephalitis	1st line: gentamicin plus ciprofloxacin	Usual dosages, i.v., 7–10 days for gentamicin and 2–4 weeks for ciprofloxacin		Very few data available. Phenicols should no longer be used, but can be discussed in case of brain involvement
	2nd line: gentamicin plus doxycycline	Usual dosages, i.v., 7–10 days for gentamicin and 2–4 weeks for doxycycline		

Clinical forms: ulceroglandular (UG), glandular (GL), oculoglandular (OG), oropharyngeal (OP), pneumonic (PN), typhoidal (TY). ($) Streptomycin can be used instead of gentamicin in countries where this antibiotic is still available although it’s usually considered more toxic.

*Antibiotic dosages are for adults. Treatment duration should be adapted to the patient status (e.g., longer in immunocompromised patients) and disease evolution. Treatment of tularemia in children should likely be similar to those for adults with appropriate dosage.

Overall, this review emphasizes the diversity and complexity of human infections with *F. tularensis*. Although antibiotic treatment is based on only three antibiotic classes, tularemia is often a chronic disease requiring several antibiotic courses combined with surgical treatment for cure. Severe forms and deep infections, although rare, may justify specific therapeutic approaches. Recommendations should be established for treatment of human tularemia cases considering the diversity of clinical symptoms, complications, and patient’s health status. It should also be stressed that tularemia diagnosis remains difficult and treatment delay of more than 2 weeks has a significant impact on antibiotic treatment efficacy and the occurrence of treatment failures, relapses, and complications. Therefore, recommendations allowing an earlier diagnosis of this disease are also essential to improve the therapeutic management of tularemia patients.

## Author contributions

MM: Conceptualization, Data curation, Formal analysis, Funding acquisition, Methodology, Supervision, Validation, Writing—original draft. LP: Data curation, Formal analysis, Investigation, Writing—original draft. AH: Data curation, Formal analysis, Investigation, Writing—original draft. IP: Data curation, Formal analysis, Investigation, Writing—original draft. SB: Data curation, Formal analysis, Investigation, Writing—original draft. YC: Conceptualization, Data curation, Formal analysis, Investigation, Writing—original draft.

## References

[B1] Ahmad S. Hunter L. Qin A. Mann B. J. van Hoek M. L. (2010). Azithromycin effectiveness against intracellular infections of *Francisella*. *BMC Microbiol.* 10:123. 10.1186/1471-2180-10-123 20416090 PMC2881020

[B2] Akdiş A. C. Kiliçturgay K. Helvaci S. Mistik R. Oral B. (1993). Immunological evaluation of erythema nodosum in tularaemia. *Br. J. Dermatol.* 129 275–279. 10.1111/j.1365-2133.1993.tb11846.x 8286224

[B3] Aloni-Grinstein R. Shifman O. Lazar S. Steinberger-Levy I. Maoz S. Ber R. (2015). A rapid real-time quantitative PCR assay to determine the minimal inhibitory extracellular concentration of antibiotics against an intracellular *Francisella tularensis* live vaccine strain. *Front. Microbiol.* 6:1213. 10.3389/fmicb.2015.01213 26579112 PMC4630301

[B4] Alqahtani M. Ma Z. Ketkar H. Suresh R. V. Malik M. Bakshi C. S. (2018). Characterization of a unique outer membrane protein required for oxidative stress resistance and virulence of *Francisella tularensis*. *J. Bacteriol.* 200 e693–e617. 10.1128/JB.00693-17 29378894 PMC5869473

[B5] Altuntas E. E. Polat K. Durmuş K. Uysal I. Ö Müderris S. (2012). Tularemia and the oculoglandular syndrome of Parinaud. *Braz. J. Infect. Dis.* 16 90–91. 22358364

[B6] Antunes N. T. Frase H. Toth M. Vakulenko S. B. (2012). The class A β-lactamase FTU-1 is native to *Francisella tularensis*. *Antimicrob. Agents Chemother.* 56 666–671. 22083489 10.1128/AAC.05305-11 PMC3264220

[B7] Arav-Boger R. (2000). Cat-bite tularemia in a seventeen-year-old girl treated with ciprofloxacin. *Pediatr. Infect. Dis. J.* 19 583–584. 10.1097/00006454-200006000-00024 10877184

[B8] Arikan O. K. Koç C. Bozdoğan O. (2003). Tularemia presenting as tonsillopharyngitis and cervical lymphadenitis: a case report and review of the literature. *Eur. Arch. Oto-Rhino-Laryngol.* 260 298–300. 10.1007/s00405-002-0565-8 12883950

[B9] Arslan F. Karagöz E. Zemheri E. Vahaboglu H. Mert A. (2016). Tick-related facial cellulitis caused by *Francisella tularensis*. *Infez. Med.* 24 140–143. 27367325

[B10] Ata N. Kılıç S. Ovet G. Alataş N. Celebi B. (2013). Tularemia during pregnancy. *Infection* 41 753–756. 23559358 10.1007/s15010-013-0456-5

[B11] Avent M. L. Rogers B. A. Cheng A. C. Paterson D. L. (2011). Current use of aminoglycosides: indications, pharmacokinetics and monitoring for toxicity. *Intern. Med. J.* 41 441–449. 21309997 10.1111/j.1445-5994.2011.02452.x

[B12] Azua E. N. Voss L. A. (2020). Tularemia in a prosthetic joint infection. *Orthopedics* 43 e54–e56. 10.3928/01477447-20190627-01 31269216

[B13] Bahuaud O. Le Brun C. Chalopin T. Lacasse M. Le Marec J. Pantaleon C. (2019). Severe infections due to *Francisella tularensis* ssp. *holarctica* in solid organ transplant recipient: report of two cases and review of literature. *BMC Infect. Dis.* 19:238. 10.1186/s12879-019-3863-0 30849949 PMC6408858

[B14] Baker C. N. Hollis D. G. Thornsberry C. (1985). Antimicrobial susceptibility testing of *Francisella tularensis* with a modified Mueller-Hinton broth. *J. Clin. Microbiol.* 22 212–215. 10.1128/jcm.22.2.212-215.1985 4031036 PMC268361

[B15] Barbaz M. Piau C. Tadie J. M. Pelloux I. Kayal S. Tattevin P. (2013). *Francisella tularensis* rhombencephalitis. *J. Clin. Microbiol.* 51 3454–3455. 23903544 10.1128/JCM.01018-13 PMC3811669

[B16] Barnes K. B. Richards M. I. Laws T. R. Núñez A. Thwaite J. E. Bentley C. (2021). Finafloxacin is an effective treatment for inhalational tularemia and plague in mouse models of infection. *Antimicrob. Agents Chemother.* 65 e2294–e2220. 10.1128/AAC.02294-20 33753342 PMC8315961

[B17] Bhatnagar N. Getachew E. Straley S. Williams J. Meltzer M. Fortier A. (1994). Reduced virulence of rifampicin-resistant mutants of *Francisella tularensis*. *J. Infect. Dis.* 170 841–847. 10.1093/infdis/170.4.841 7930725

[B18] Bina X. R. Lavine C. L. Miller M. A. Bina J. E. (2008). The AcrAB RND efflux system from the live vaccine strain of *Francisella tularensis* is a multiple drug efflux system that is required for virulence in mice. *FEMS Microbiol. Lett.* 279 226–233. 10.1111/j.1574-6968.2007.01033.x 18179581

[B19] Bina X. R. Wang C. Miller M. A. Bina J. E. (2006). The Bla2 beta-lactamase from the live-vaccine strain of *Francisella tularensis* encodes a functional protein that is only active against penicillin-class beta-lactam antibiotics. *Arch. Microbiol.* 186 219–228. 10.1007/s00203-006-0140-6 16841206

[B20] Binay U. D. Barkay O. Karakeçili F. (2023). Tularemia in the differential diagnosis of lymphadenitis: a retrospective analysis of 16 cases. *Cureus* 15:e38920. 10.7759/cureus.38920 37309344 PMC10257801

[B21] Biot F. V. Bachert B. A. Mlynek K. D. Toothman R. G. Koroleva G. I. Lovett S. P. (2020). Evolution of antibiotic resistance in surrogates of *Francisella tularensis* (LVS and *Francisella novicida*): effects on biofilm formation and fitness. *Front. Microbiol.* 11:593542. 10.3389/fmicb.2020.593542 33193267 PMC7661474

[B22] Bishop A. Wang H.-H. Donaldson T. G. Brockinton E. E. Kothapalli E. Clark S. (2023). Tularemia cases increase in the USA from 2011 through 2019. *Curr. Res. Parasitol. Vector-Borne Dis.* 3:100116. 10.1016/j.crpvbd.2023.100116 PMC9972391 36865594

[B23] Biswas S. Raoult D. Rolain J.-M. (2008). A bioinformatic approach to understanding antibiotic resistance in intracellular bacteria through whole genome analysis. *Int. J. Antimicrob. Agents* 32 207–220. 18619818 10.1016/j.ijantimicag.2008.03.017

[B24] Boone I. Hassler D. Nguyen T. Splettstoesser W. D. Wagner-Wiening C. Pfaff G. (2015). Tularaemia in southwest Germany: three cases of tick-borne transmission. *Ticks Tick-Borne Dis.* 6 611–614. 10.1016/j.ttbdis.2015.05.004 26055233

[B25] Bossi P. Tegnell A. Baka A. Van Loock F. Hendriks J. Werner A. (2004). Bichat guidelines for the clinical management of tularaemia and bioterrorism-related tularaemia. *Euro Surveill.* 9 E9–E10. 15677845

[B26] Bowe D. P. Wakeman D. C. (1936). Tularemia and pregnancy: report of a case. *J. Am. Med. Assoc.* 107 577–578.

[B27] Bricker D. (1931). Tularemia Infection during Pregnancy. *Am. J. Nurs.* 31 979–982.

[B28] Briere M. Kaladji A. Douane F. Breux J. P. Touroult-Jupin P. Boisset S. (2016). *Francisella tularensis* aortitis. *Infection* 44 263–265. 10.1007/s15010-015-0824-4 26189939

[B29] Cağlı S. Vural A. Sönmez O. Yüce I. Güney E. (2011). Tularemia: a rare cause of neck mass, evaluation of 33 patients. *Eur. Arch. Oto-Rhino-Laryngol.* 268 1699–1704. 10.1007/s00405-011-1722-8 21814733

[B30] Calin R. Caumes E. Reibel F. Ali Mohamed A. Brossier F. Foltz V. (2017). Severe glandular tularemia in a patient treated with anti-tumour necrosis factor for psoriatic arthritis. *Int. J. Infect. Dis.* 60 1–3. 28450199 10.1016/j.ijid.2017.04.014

[B31] Caspar Y. Hennebique A. Maurin M. (2018). Antibiotic susceptibility of *Francisella tularensis* subsp. *holarctica* strains isolated from tularaemia patients in France between 2006 and 2016. *J. Antimicrob. Chemother.* 73 687–691. 10.1093/jac/dkx460 29253157

[B32] Caspar Y. Maurin M. (2017). *Francisella tularensis* susceptibility to antibiotics: a comprehensive review of the data obtained in vitro and in animal models. *Front. Cell. Infect. Microbiol.* 7:122. 10.3389/fcimb.2017.00122 28443249 PMC5386985

[B33] Caspar Y. Siebert C. Sutera V. Villers C. Aubry A. Mayer C. (2017). Functional Characterization of the DNA gyrases in Fluoroquinolone-resistant Mutants of *Francisella* novicida. *Antimicrob. Agents Chemother.* 61 e2277–e2216. 10.1128/AAC.02277-16 28167561 PMC5365679

[B34] Caspar Y. Sutera V. Boisset S. Denis J.-N. Maurin M. (2014). Bis-indolic compounds as potential new therapeutic alternatives for tularaemia. *Front. Cell. Infect. Microbiol.* 4:24. 10.3389/fcimb.2014.00024 24579066 PMC3936198

[B35] CDC | Bioterrorism Agents/Diseases (by category) | Emergency Preparedness & Response (2019). Available online at: https://emergency.cdc.gov/agent/agentlist-category.asp (accessed 6 April, 2023).

[B36] Celebi G. Baruönü F. Ayoğlu F. Cinar F. Karadenizli A. Uğur M. B. (2006). Tularemia, a reemerging disease in northwest Turkey: epidemiological investigation and evaluation of treatment responses. *Jpn. J. Infect. Dis.* 59 229–234. 16936340

[B37] Celik T. Kosker M. Kirboga K. (2014). An atypical case of tularemia presented with pseudoptosis. *Infection* 42 785–788. 10.1007/s15010-014-0613-5 24659144

[B38] Celik T. Kosker M. Turkoglu E. B. (2013). Unilateral acute dacryocystitis associated with oculoglandular tularemia: a case report. *Semin. Ophthalmol.* 28 91–93. 10.3109/08820538.2012.760624 23448564

[B39] Centers for Disease Control and Prevention (CDC) (2009). Tularemia - Missouri, 2000-2007. *MMWR Morb. Mortal. Wkly. Rep.* 58 744–748. 19609248

[B40] Cerný Z. (1994). Skin manifestations of tularemia. *Int. J. Dermatol.* 33 468–470. 7928027 10.1111/j.1365-4362.1994.tb02855.x

[B41] Charles P. Stumpf P. Buffet P. Hot A. Lecuit M. Dupont B. (2008). Two unusual glandular presentations of tick-borne tularemia. *Médecine Mal. Infect.* 38 159–161. 10.1016/j.medmal.2007.11.005 18191519

[B42] Chitadze N. Kuchuloria T. Clark D. V. Tsertsvadze E. Chokheli M. Tsertsvadze N. (2009). Water-borne outbreak of oropharyngeal and glandular tularemia in Georgia: investigation and follow-up. *Infection* 37 514–521. 10.1007/s15010-009-8193-5 19826763

[B43] Chrdle A. Trnka T. Musil D. Fucentese S. F. Bode P. Keller P. M. (2019). *Francisella tularensis* periprosthetic joint infections diagnosed with growth in cultures. *J. Clin. Microbiol.* 57 e339–e319. 10.1128/JCM.00339-19 31189580 PMC6663894

[B44] Clary S. J. Brubacher J. W. Kubat R. C. (2022). Tularemia proximal interphalangeal joint septic arthritis: a case report. *JBJS Case Connect.* 12:e22.00287. 10.2106/JBJS.CC.22.00287 36049027

[B45] Clinical And Laboratory Standards Institute (2005). *CLSI M45-Ed1 - Methods for Antimicrobial Dilution and Disk Susceptibility Testing of Infrequently Isolated or Fastidious Bacteria, 1st Edition.* Available online at: https://infostore.saiglobal.com/en-us/Standards/CLSI-M45-P-1ED-2005-357801_SAIG_CLSI_CLSI_814931/ (accessed 8 December, 2022).

[B46] Clinical And Laboratory Standards Institute (2016). *CLSI M45-Ed3 - Methods for Antimicrobial Dilution and Disk Susceptibility Testing of Infrequently Isolated or Fastidious Bacteria, 3rd Edition.* Available online at: https://webstore.ansi.org/standards/clsi/clsim45ed3 (accessed 8 December, 2022).

[B47] Çoban E. Serindağ H. C. Kara E. S. Selçuk H. H. Eren F. Albay V. B. (2021). Central nervous system vasculitis due to an endemic zoonosis in Turkey. *Tularemia. Noro Psikiyatri Arsivi* 58 73–76. 10.29399/npa.23491 33795957 PMC7980708

[B48] Contentin L. Soret J. Zamfir O. Gontier O. Lherm T. Hamrouni M. (2011). *Francisella tularensis* meningitis. *Médecine Mal. Infect.* 41 556–558. 10.1016/j.medmal.2011.07.004 21831551

[B49] Cooper C. L. Van Caeseele P. Canvin J. Nicolle L. E. (1999). Chronic prosthetic device infection with *Francisella tularensis*. *Clin. Infect. Dis.* 29 1589–1591. 10.1086/313550 10585830

[B50] Crane D. D. Scott D. P. Bosio C. M. (2012). Generation of a convalescent model of virulent *Francisella tularensis* infection for assessment of host requirements for survival of tularemia. *PLoS One* 7:e33349. 10.1371/journal.pone.0033349 22428026 PMC3299770

[B51] Cross J. T. Jr. Schutze G. E. Jacobs R. F. (1995). Treatment of tularemia with gentamicin in pediatric patients. *Pediatr. Infect. Dis. J.* 14 151–152. 7746700

[B52] Darmon-Curti A. Darmon F. Edouard S. Hennebique A. Guimard T. Martin-Blondel G. (2020). Tularemia: a case series of patients diagnosed at the national reference center for Rickettsioses from 2008 to 2017. *Open Forum Infect. Dis.* 7:ofaa440. 10.1093/ofid/ofaa440 33209946 PMC7651688

[B53] Dennis D. T. Inglesby T. V. Henderson D. A. Bartlett J. G. Ascher M. S. Eitzen E. (2001). Tularemia as a biological weapon: medical and public health management. *JAMA J. Am. Med. Assoc.* 285 2763–2773. 10.1001/jama.285.21.2763 11386933

[B54] Dentan C. Pavese P. Pelloux I. Boisset S. Brion J.-P. Stahl J.-P. (2013). Treatment of tularemia in pregnant woman, france. *Emerg. Infect. Dis.* 19 996–998. 23735285 10.3201/eid1906.130138 PMC3713841

[B55] Di Ninno V. L. Cherwanogrodzky J. W. Wong J. P. (1993). Liposome-encapsulated ciprofloxacin is effective in the protection and treatment of BALB/c mice against *Francisella tularensis*. *J. Infect. Dis.* 168 793–794. 10.1093/infdis/168.3.793 8354928

[B56] Dryselius R. Hjertqvist M. Mäkitalo S. Lindblom A. Lilja T. Eklöf D. (2019). Large outbreak of tularaemia, central Sweden. July to September 2019. *Euro Surveill.* 24:1900603. 10.2807/1560-7917.ES.2019.24.42.1900603 31640844 PMC6807254

[B57] Ducatez N. Melboucy S. Bentayeb H. Dayen C. Suguenot R. Lecuyer E. (2022). A case of *Francisella tularensis* meningitis in a 64-year-old man treated with quinolones. *Infect. Dis. Now* 52 107–109. 10.1016/j.idnow.2021.06.306 34242839

[B58] Eliasson H. Bäck E. (2007). Tularaemia in an emergent area in Sweden: an analysis of 234 cases in five years. *Scand. J. Infect. Dis.* 39 880–889. 10.1080/00365540701402970 17886125

[B59] Eliopoulos G. M. Drusano G. L. Ambrose P. G. Bhavnani S. M. Bertino J. S. Nafziger A. N. (2007). Back to the future: using aminoglycosides again and how to dose them optimally. *Clin. Infect. Dis.* 45 753–760. 10.1086/520991 17712761

[B60] Ellis J. Oyston P. C. F. Green M. Titball R. W. (2002). Tularemia. *Clin. Microbiol. Rev.* 15 631–646. 12364373 10.1128/CMR.15.4.631-646.2002 PMC126859

[B61] Erdem H. Ozturk-Engin D. Yesilyurt M. Karabay O. Elaldi N. Celebi G. (2014). Evaluation of tularaemia courses: a multicentre study from Turkey. *Clin. Microbiol. Infect.* 20 O1042–O1051. 10.1111/1469-0691.12741 24975504

[B62] Eren Gok S. Kocagul Celikbas A. Baykam N. Atay Buyukdemirci A. Eroglu M. N. Evren Kemer O. (2014). Evaluation of tularemia cases focusing on the oculoglandular form. *J. Infect. Dev. Ctries.* 8 1277–1284. 10.3855/jidc.3996 25313604

[B63] Escovar J. Patil S. M. Roland W. (2023). Acute left knee prosthetic joint infection by *Francisella tularensis* with literature review. *IDCases* 33:e01812. 10.1016/j.idcr.2023.e01812 37645536 PMC10461113

[B64] Esmaeili S. Rohani M. Ghasemi A. Gouya M. M. Khayatzadeh S. Mahmoudi A. (2021). *Francisella tularensis* human infections in a village of northwest Iran. *BMC Infect. Dis.* 21:310. 10.1186/s12879-021-06004-y 33789598 PMC8010941

[B65] Evans M. E. Gregory D. W. Schaffner W. McGee Z. A. (1985). Tularemia: a 30-year experience with 88 cases. *Medicine (Baltimore)* 64 251–269. 3892222

[B66] Fachinger P. Tini G. M. Grobholz R. Gambazzi F. Fankhauser H. Irani S. (2015). Pulmonary tularaemia: all that looks like cancer is not necessarily cancer - case report of four consecutive cases. *BMC Pulm. Med.* 15:27. 10.1186/s12890-015-0026-y 25887439 PMC4386093

[B67] Faucon A.-L. Zamfir O. Dupouët L. Pruna A. (2011). *Streptococcus pneumonia* infection and positive blood culture with *Francisella tularensis*, in a renal transplant recipient. *Presse Med.* 1983 1199–1202. 10.1016/j.lpm.2011.05.017 21816564

[B68] Feldman K. A. Enscore R. E. Lathrop S. L. Matyas B. T. McGuill M. Schriefer M. E. (2001). An outbreak of primary pneumonic tularemia on Martha’s Vineyard. *N. Engl. J. Med.* 345 1601–1606. 11757506 10.1056/NEJMoa011374

[B69] Fiscella R. G. Gieser J. Phillpotts B. Gilmartin C. Labib S. Cwik M. J. (1998). Intraocular penetration of gentamicin after once-daily aminoglycoside dosing. *Retina* 18 339–342. 10.1097/00006982-199807000-00008 9730177

[B70] Formińska K. Zasada A. A. Rastawicki W. Śmietańska K. Bander D. Wawrzynowicz-Syczewska M. (2015). Increasing role of arthropod bites in tularaemia transmission in Poland - case reports and diagnostic methods. *Ann. Agric. Environ. Med. AAEM* 22 443–446. 10.5604/12321966.1167711 26403111

[B71] Frischknecht M. Meier A. Mani B. Joerg L. Kim O. C.-H. Boggian K. (2019). Tularemia: an experience of 13 cases including a rare myocarditis in a referral center in Eastern Switzerland (Central Europe) and a review of the literature. *Infection* 47 683–695. 10.1007/s15010-019-01269-7 30656604

[B72] Fritzsch J. Splettstoesser W. D. (2010). Septic pneumonic tularaemia caused by *Francisella tularensis* subsp. *holarctica* biovar II. *J. Med. Microbiol.* 59 1123–1125. 10.1099/jmm.0.019893-0 20522628

[B73] Gaci R. Alauzet C. Selton-Suty C. Lozniewski A. Pulcini C. May T. (2017). *Francisella tularensis* endocarditis: two case reports and a literature review. *Infect. Dis. Lond. Engl.* 49 128–131. 10.1080/23744235.2016.1222546 27564142

[B74] Gangat N. (2007). Cerebral abscesses complicating tularemia meningitis. *Scand. J. Infect. Dis.* 39 258–261. 10.1080/00365540600823243 17366059

[B75] Garver M. K. St Geme J. W. III Siegel M. J. (1994). Tularemia presenting with splenic nodules. *Pediatr. Infect. Dis. J.* 13 830–832. 10.1097/00006454-199409000-00018 7808857

[B76] Georgi E. Schacht E. Scholz H. C. Splettstoesser W. D. (2012). Standardized broth microdilution antimicrobial susceptibility testing of *Francisella tularensis* subsp. *holarctica* strains from Europe and rare *Francisella* species. *J. Antimicrob. Chemother.* 67 2429–2433. 10.1093/jac/dks238 22763567

[B77] Gestin B. Valade E. Thibault F. Schneider D. Maurin M. (2010). Phenotypic and genetic characterization of macrolide resistance in *Francisella tularensis* subsp. *holarctica* biovar I. *J. Antimicrob. Chemother.* 65 2359–2367. 10.1093/jac/dkq315 20837574

[B78] Gil H. Platz G. J. Forestal C. A. Monfett M. Bakshi C. S. Sellati T. J. (2006). Deletion of TolC orthologs in *Francisella tularensis* identifies roles in multidrug resistance and virulence. *Proc. Natl. Acad. Sci. U. S. A.* 103 12897–12902. 10.1073/pnas.0602582103 16908853 PMC1568944

[B79] Gill V. Cunha B. A. (1997). Tularemia pneumonia. *Semin. Respir. Infect.* 12 61–67. 9097380

[B80] Glynn A. R. Alves D. A. Frick O. Erwin-Cohen R. Porter A. Norris S. (2015). Comparison of experimental respiratory tularemia in three nonhuman primate species. *Comp. Immunol. Microbiol. Infect. Dis.* 39 13–24. 10.1016/j.cimid.2015.01.003 25766142 PMC4397973

[B81] Gönen İ (2013). A small outbreak of tularemia in a rural area. *Turk. J. Med. Sci.* 43 75–78.

[B82] Gozel M. G. Engin A. Altuntas E. E. Salk İ Kaya A. Celik C. (2014). Evaluation of clinical and laboratory findings of pediatric and adult patients with oropharyngeal tularemia in Turkey: a combination of surgical drainage and antibiotic therapy increases treatment success. *Jpn. J. Infect. Dis.* 67 295–299. 10.7883/yoken.67.295 25056077

[B83] Grossman T. H. Anderson M. S. Christ D. Gooldy M. Henning L. N. Heine H. S. (2017). The Fluorocycline TP-271 is efficacious in models of aerosolized *Francisella tularensis* SCHU S4 Infection in BALB/c mice and Cynomolgus Macaques. *Antimicrob. Agents Chemother.* 61:e448-17. 10.1128/AAC.00448-17 28559261 PMC5527625

[B84] Guerpillon B. Boibieux A. Guenne C. Ploton C. Ferry T. Maurin M. (2016). Keep an ear out for *Francisella tularensis*: otomastoiditis cases after canyoneering. *Front. Med.* 3:9. 10.3389/fmed.2016.00009 26973838 PMC4776157

[B85] Gürkov R. Kisser U. Splettstösser W. Hogardt M. Krause E. (2009). Tularaemia of middle ear with suppurative lymphadenopathy and retropharyngeal abscess. *J. Laryngol. Otol.* 123 1252–1257. 10.1017/S0022215109004757 19250590

[B86] Hahn M. M. Triplett C. A. Anderson M. S. Smart J. I. Litherland K. Keech S. (2023). Ceftobiprole Medocaril is an effective post-exposure treatment in the fischer 344 rat model of Pneumonic Tularemia. *Antibiot. Basel Switz.* 12:1337. 10.3390/antibiotics12081337 37627757 PMC10451734

[B87] Hamblin K. A. Wong J. P. Blanchard J. D. Atkins H. S. (2014). The potential of liposome-encapsulated ciprofloxacin as a tularemia therapy. *Front. Cell. Infect. Microbiol.* 4:79. 10.3389/fcimb.2014.00079 24995163 PMC4062069

[B88] Han X. Y. Ho L. X. Safdar A. (2004). *Francisella tularensis* peritonitis in stomach cancer patient. *Emerg. Infect. Dis.* 10 2238–2240. 10.3201/eid1012.040497 15663872 PMC3323394

[B89] Haulrig M. B. Mathiasen G. Nielsen R. M. Kromann C. B. Krogfelt K. A. Wiese L. (2020). Two cases of tick-borne transmitted tularemia on Southern Zealand. *Denmark. APMIS* 128 61–64. 10.1111/apm.13008 31691353

[B90] Hauri A. M. Hofstetter I. Seibold E. Kaysser P. Eckert J. Neubauer H. (2010). Investigating an airborne tularemia outbreak, Germany. *Emerg. Infect. Dis.* 16 238–243. 10.3201/eid1602.081727 20113553 PMC2957990

[B91] Heine H. S. Miller L. Halasohoris S. Purcell B. K. (2017). In vitro antibiotic susceptibilities of *Francisella tularensis* determined by broth microdilution following CLSI methods. *Antimicrob. Agents Chemother.* 61 e612–e617. 10.1128/AAC.00612-17 28674048 PMC5571322

[B92] Helvaci S. Gedikoğlu S. Akalin H. Oral H. B. (2000). Tularemia in Bursa, Turkey: 205 cases in ten years. *Eur. J. Epidemiol.* 16 271–276. 10.1023/a:1007610724801 10870943

[B93] Hennebique A. Boisset S. Maurin M. (2019). Tularemia as a waterborne disease: a review. *Emerg. Microbes Infect.* 8 1027–1042. 31287787 10.1080/22221751.2019.1638734 PMC6691783

[B94] Hepburn M. J. Simpson A. J. H. (2008). Tularemia: current diagnosis and treatment options. *Expert Rev. Anti. Infect. Ther.* 6 231–240. 18380605 10.1586/14787210.6.2.231

[B95] Hofinger D. M. Cardona L. Mertz G. J. Davis L. E. (2009). Tularemic meningitis in the United States. *Arch. Neurol.* 66 523–527. 19364939 10.1001/archneurol.2009.14

[B96] Holland S. D. Michelow I. C. (2016). Tularemia Masquerading as Ecthyma. *J. Pediatr.* 178:299. 10.1016/j.jpeds.2016.07.023 27562918

[B97] Hotta A. Fujita O. Uda A. Sharma N. Tanabayashi K. Yamamoto Y. (2013). In vitro antibiotic susceptibility of *Francisella tularensis* isolates from Japan. *Jpn. J. Infect. Dis.* 66 534–536. 10.7883/yoken.66.534 24270145

[B98] Ikäheimo I. Syrjälä H. Karhukorpi J. Schildt R. Koskela M. (2000). In vitro antibiotic susceptibility of *Francisella tularensis* isolated from humans and animals. *J. Antimicrob. Chemother.* 46 287–290. 10.1093/jac/46.2.287 10933655

[B99] Jackson J. McGregor A. Cooley L. Ng J. Brown M. Ong C. W. (2012). *Francisella tularensis* subspecies *holarctica*, Tasmania, Australia, 2011. *Emerg. Infect. Dis.* 18 1484–1486. 10.3201/eid1809.111856 22931809 PMC3437722

[B100] Jaing C. J. McLoughlin K. S. Thissen J. B. Zemla A. Gardner S. N. Vergez L. M. (2016). Identification of genome-wide mutations in ciprofloxacin-resistant *F. tularensis* LVS using whole genome tiling arrays and next generation sequencing. *PLoS One* 11:e0163458. 10.1371/journal.pone.0163458 27668749 PMC5036845

[B101] Johansson A. Berglund L. Gothefors L. Sjöstedt A. Tärnvik A. (2000). Ciprofloxacin for treatment of tularemia in children. *Pediatr. Infect. Dis. J.* 19 449–453. 10819342 10.1097/00006454-200005000-00011

[B102] Johansson A. Berglund L. Sjöstedt A. Tärnvik A. (2001). Ciprofloxacin for treatment of tularemia. *Clin. Infect. Dis.* 33 267–268. 11418893 10.1086/321825

[B103] Johansson A. Farlow J. Larsson P. Dukerich M. Chambers E. Byström M. (2004). Worldwide genetic relationships among *Francisella tularensis* isolates determined by multiple-locus variable-number tandem repeat analysis. *J. Bacteriol.* 186 5808–5818. 10.1128/JB.186.17.5808-5818.2004 15317786 PMC516809

[B104] Johansson A. Lärkeryd A. Widerström M. Mörtberg S. Myrtännäs K. Ohrman C. (2014). A respiratory tularemia outbreak caused by diverse clones of *Francisella tularensis*. *Clin. Infect. Dis.* 59 1546–1553. 10.1093/cid/ciu621 25097081 PMC4650766

[B105] Johansson A. Urich S. K. Chu M. C. Sjöstedt A. Tärnvik A. (2002). In vitro susceptibility to quinolones of *Francisella tularensis* subspecies *tularensis*. *Scand. J. Infect. Dis.* 34 327–330. 12069013 10.1080/00365540110080773

[B106] Johnsrud J. J. Smith C. R. Bradsher R. W. (2019). Serendipitous treatment of Tularemia in pregnancy. *Open Forum Infect. Dis.* 6:ofz413. 10.1093/ofid/ofz413 31660371 PMC6804751

[B107] Junkins A. D. Snyder J. W. (2011). Cellulitis, headache, and fever following tick bites. *J. Clin. Microbiol.* 49 2085–2387. 10.1128/JCM.00075-11 21617001 PMC3122739

[B108] Kaeppler M. Kapoor R. Shah N. Katbamna B. Wantz M. Kott A. (2020). Tick-borne illness and infective endocarditis: a rare case of Tularemia. *CASE* 4 78–81. 10.1016/j.case.2019.10.002 32337395 PMC7175796

[B109] Kantardjiev T. Ivanov I. Velinov T. Padeshki P. Popov B. Nenova R. (2006). Tularemia outbreak, Bulgaria, 1997-2005. *Emerg. Infect. Dis.* 12 678–680. 16704820 10.3201/eid1204.050709 PMC3294687

[B110] Karakas A. Coskun O. Artuk C. Savasci U. Gul H. C. Mert G. (2014). Oropharyngeal tularemia cases admitted to a military hospital in Ankara. *Turkey. J. Infect. Dev. Ctries.* 8 994–999. 10.3855/jidc.4332 25116664

[B111] Karlı A. Şensoy G. Paksu Ş Korkmaz M. F. Ertuğrul Ö Karlı R. (2018). Treatment-failure tularemia in children. *Korean J. Pediatr.* 61 49–52. 29563944 10.3345/kjp.2018.61.2.49 PMC5854842

[B112] Karlsson E. Golovliov I. Lärkeryd A. Granberg M. Larsson E. Öhrman C. (2016). Clonality of erythromycin resistance in *Francisella tularensis*. *J. Antimicrob. Chemother.* 71 2815–2823. 10.1093/jac/dkw235 27334667

[B113] Kassinger S. J. van Hoek M. L. (2021). Genetic determinants of antibiotic resistance in *Francisella*. *Front. Microbiol.* 12:644855. 10.3389/fmicb.2021.644855 34054749 PMC8149597

[B114] Kaya A. Uysal I. Ö Güven A. S. Engin A. Gültürk A. İçağasıoğlu F. D. (2011). Treatment failure of gentamicin in pediatric patients with oropharyngeal tularemia. *Med. Sci. Monit. Int. Med. J. Exp. Clin. Res.* 17 CR376–CR380. 10.12659/MSM.881848 PMC3539565 21709631

[B115] Khoury J. A. Bohl D. L. Hersh M. J. Argoudelis A. C. Brennan D. C. (2005). Tularemia in a kidney transplant recipient: an unsuspected case and literature review. *Am. J. Kidney Dis.* 45 926–929. 10.1053/j.ajkd.2005.02.006 15861359

[B116] Kiliç S. Celebi B. Acar B. Ataş M. (2013). In vitro susceptibility of isolates of *Francisella tularensis* from Turkey. *Scand. J. Infect. Dis.* 45 337–341. 23249114 10.3109/00365548.2012.751125

[B117] Kızıl Y. Aydil U. Cebeci S. Güzeldir O. T. Inal E. Bayazıt Y. (2012). Characteristics and management of intractable neck involvement in tularemia: report of 19 patients. *Eur. Arch. Oto-Rhino-Laryngol.* 269 1285–1290. 10.1007/s00405-011-1830-5 22068840

[B118] Klimpel G. R. Eaves-Pyles T. Moen S. T. Taormina J. Peterson J. W. Chopra A. K. (2008). Levofloxacin rescues mice from lethal intra-nasal infections with virulent *Francisella tularensis* and induces immunity and production of protective antibody. *Vaccine* 26 6874–6882. 10.1016/j.vaccine.2008.09.077 18930100 PMC2630585

[B119] Kocabaş E. Özgür Gündeşlioğlu Ö Kılıç Çil M. Çay Ü Doran F. Soyupak S. (2020). A rare cause of granulomatous hepatitis: Tularemia. *J. Infect. Public Health* 13 1003–1005. 10.1016/j.jiph.2019.12.007 31937491

[B120] Komitova R. Nenova R. Padeshki P. Ivanov I. Popov V. Petrov P. (2010). Tularemia in bulgaria 2003-2004. *J. Infect. Dev. Ctries.* 4 689–694. 10.3855/jidc.712 21252445

[B121] Köse H. C. Hoşal M. B. (2019). A rare complication of oropharyngeal Tularemia: Dacryocystitis. *Turk. J. Ophthalmol.* 49 164–167. 10.4274/tjo.galenos.2018.96337 31245979 PMC6624460

[B122] Kosker M. Sener D. Kilic O. Akil F. Yilmaz M. Ozturk O. (2013). A case of oculoglandular tularemia resistant to medical treatment. *Scand. J. Infect. Dis.* 45 725–727. 10.3109/00365548.2013.796089 23746340

[B123] Kreizinger Z. Makrai L. Helyes G. Magyar T. Erdélyi K. Gyuranecz M. (2013). Antimicrobial susceptibility of *Francisella tularensis* subsp. *holarctica* strains from Hungary, Central Europe. *J. Antimicrob. Chemother.* 68 370–373. 10.1093/jac/dks399 23065699

[B124] Kreutzmann T. Schönfeld A. Zange S. Lethaus B. (2021). A case report of oculoglandular tularemia-chasing zebras among potential diagnoses. *J. Oral Maxillofac. Surg.* 79 629–636. 10.1016/j.joms.2020.08.018 32949503

[B125] Kubiliute I. Zablockiene B. Paulauskiene R. Navickas G. Jancoriene L. (2021). A Rare case of tularemia complicated by rhabdomyolysis with a successful outcome. *Med. Kaunas Lith.* 57:449. 10.3390/medicina57050449 34062973 PMC8147915

[B126] Kulshrestha O. P. Kurian Keshwan H. H. R. (1981). Penetration of doxycycline in aqueous humour. *Indian J. Ophthalmol.* 29:251. 7346437

[B127] Kuzmova M. Rondelet B. Belhaj A. (2023). A rare case of aortic endograft infection by *Francisella tularensis*: a case report. *Int. J. Surg. Case Rep.* 110:108685. 10.1016/j.ijscr.2023.108685 37634431 PMC10509798

[B128] La Scola B. Elkarkouri K. Li W. Wahab T. Fournous G. Rolain J.-M. (2008). Rapid comparative genomic analysis for clinical microbiology: the *Francisella tularensis* paradigm. *Genome Res.* 18 742–750. 10.1101/gr.071266.107 18407970 PMC2336804

[B129] Landais C. Levy P.-Y. Habib G. Raoult D. (2008). Pericardial effusion as the only manifestation of infection with *Francisella tularensis*: a case report. *J. Med. Case Reports* 2:206. 10.1186/1752-1947-2-206 18554395 PMC2441635

[B130] Larssen K. W. Afset J. E. Heier B. T. Krogh T. Handeland K. Vikøren T. (2011). Outbreak of tularaemia in central Norway, January to March 2011. *Euro Surveill.* 16:19828. 21489376

[B131] Li Y. Powell D. A. Shaffer S. A. Rasko D. A. Pelletier M. R. Leszyk J. D. (2012). LPS remodeling is an evolved survival strategy for bacteria. *Proc. Natl. Acad. Sci. U. S. A.* 109 8716–8721. 10.1073/pnas.1202908109 22586119 PMC3365160

[B132] Limaye A. P. Hooper C. J. (1999). Treatment of tularemia with fluoroquinolones: two cases and review. *Clin. Infect. Dis.* 29 922–924. 10589911 10.1086/520458

[B133] Lovell V. M. Cho C. T. Lindsey N. J. Nelson P. L. (1986). *Francisella tularensis* meningitis: a rare clinical entity. *J. Infect. Dis.* 154 916–918. 10.1093/infdis/154.5.916 3772171

[B134] LoVullo E. D. Miller C. N. Pavelka M. S. Kawula T. H. (2012). TetR-based gene regulation systems for *Francisella tularensis*. *Appl. Environ. Microbiol.* 78 6883–6889. 10.1128/AEM.01679-12 22820330 PMC3457491

[B135] Ma Z. Banik S. Rane H. Mora V. T. Rabadi S. M. Doyle C. R. (2014). EmrA1 membrane fusion protein of *Francisella tularensis* LVS is required for resistance to oxidative stress, intramacrophage survival and virulence in mice. *Mol. Microbiol.* 91 976–995. 10.1111/mmi.12509 24397487 PMC4097035

[B136] Madrid P. B. Chopra S. Manger I. D. Gilfillan L. Keepers T. R. Shurtleff A. C. (2013). A systematic screen of FDA-approved drugs for inhibitors of biological threat agents. *PLoS One* 8:e60579. 10.1371/journal.pone.0060579 23577127 PMC3618516

[B137] Mahy S. Chavanet P. Piroth L. Roch N. Duong M. Pelloux I. (2011). Emergence of tularemia in France: paradigm of the Burgundy region. *Int. J. Infect. Dis.* 15 e882–e883. 10.1016/j.ijid.2011.08.007 21975180

[B138] Marquart J. D. Clifford R. (2015). Pneumonic tularemia presenting with a vesicular eruption. *Cutis* 95 E17–E18. 25942033

[B139] Martinet P. Khatchatourian L. Saidani N. Fangous M.-S. Goulon D. Lesecq L. (2021). Hypermetabolic pulmonary lesions on FDG-PET/CT: Tularemia or neoplasia? *Infect. Dis. Now* 51 607–613. 34242840 10.1016/j.idnow.2021.06.307

[B140] Martínez-Martínez S. Rodríguez-Ferri E.-F. Rodríguez-Lázaro D. Hernández M. Gómez-Campillo J.-I. Martínez-Nistal M. D. C. (2021). In vitro antimicrobial susceptibilities of *Francisella tularensis* subsp. *holarctica* isolates from Tularemia outbreaks that occurred from the end of the 20th century to the 2020s in Spain. *Antibiot. Basel Switz.* 10:938. 10.3390/antibiotics10080938 34438988 PMC8389022

[B141] Matyas B. T. Nieder H. S. Telford S. R. (2007). Pneumonic tularemia on Martha’s Vineyard: clinical, epidemiologic, and ecological characteristics. *Ann. N. Y. Acad. Sci.* 1105 351–377. 10.1196/annals.1409.013 17442781

[B142] Maurin M. (2020). *Francisella tularensis*, Tularemia and serological diagnosis. *Front. Cell. Infect. Microbiol.* 10:512090. 10.3389/fcimb.2020.512090 33194778 PMC7649319

[B143] Maurin M. Gyuranecz M. (2016). Tularaemia: clinical aspects in Europe. *Lancet Infect. Dis.* 16 113–124. 26738841 10.1016/S1473-3099(15)00355-2

[B144] Maurin M. Mersali N. F. Raoult D. (2000). Bactericidal activities of antibiotics against intracellular *Francisella tularensis*. *Antimicrob. Agents Chemother.* 44 3428–3431. 10.1128/AAC.44.12.3428-3431.2000 11083651 PMC90216

[B145] Maurin M. Pelloux I. Brion J. P. Del Banõ J.-N. Picard A. (2011). Human tularemia in France, 2006-2010. *Clin. Infect. Dis.* 53 e133–e141. 10.1093/cid/cir612 22002987

[B146] Mehta H. H. Ibarra D. Marx C. J. Miller C. R. Shamoo Y. (2022). Mutational switch-backs can accelerate evolution of *Francisella* to a combination of Ciprofloxacin and Doxycycline. *Front. Microbiol.* 13:904822. 10.3389/fmicb.2022.904822 35615518 PMC9125183

[B147] Meric M. Willke A. Finke E.-J. Grunow R. Sayan M. Erdogan S. (2008). Evaluation of clinical, laboratory, and therapeutic features of 145 tularemia cases: the role of quinolones in oropharyngeal tularemia. *APMIS* 116 66–73. 10.1111/j.1600-0463.2008.00901.x 18254782

[B148] Mittalhenkle A. Norman D. J. (2006). Tularemia in a renal transplant recipient. *Clin. Transpl.* 574–575. 18365440

[B149] Morlet N. Graham G. G. Gatus B. McLachlan A. J. Salonikas C. Naidoo D. (2000). Pharmacokinetics of Ciprofloxacin in the human eye: a clinical study and population Pharmacokinetic analysis. *Antimicrob. Agents Chemother.* 44 1674–1679. 10.1128/AAC.44.6.1674-1679.2000 10817727 PMC89931

[B150] Mounier M. Adenis J. P. Denis F. (1988). Intraocular penetration of ciprofloxacin after infusion and oral administration. *Pathol. Biol.* 36 724–727. 3054755

[B151] Nakamura K. Fujita H. Miura T. Igata Y. Narita M. Monma N. (2018). A case of typhoidal tularemia in a male Japanese farmer. *Int. J. Infect. Dis.* 71 56–58. 10.1016/j.ijid.2018.03.023 29635071

[B152] Naughton M. Brown R. Adkins D. DiPersio J. (1999). Tularemia – an unusual cause of a solitary pulmonary nodule in the post-transplant setting. *Bone Marrow Transplant.* 24 197–199. 10.1038/sj.bmt.1701863 10455349

[B153] Nemmour A. Bakri A. Fischer C. A. Brand Y. (2019). Paediatric oropharyngeal tularaemia requiring surgical intervention. *BMJ Case Rep.* 12:e229754. 10.1136/bcr-2019-229754 PMC6731923 31494583

[B154] Olivo C. A. Dysart C. Haque J. Olivo C. El-Dalati S. Gundacker N. (2019). A rare cause of prosthetic valve infective Endocarditis: *Francisella tularensis holarctica*. *WMJ* 118 196–198. 31978290

[B155] Origgi F. C. Frey J. Pilo P. (2014). Characterisation of a new group of *Francisella tularensis* subsp. *holarctica* in Switzerland with altered antimicrobial susceptibilities, 1996 to 2013. *Euro Surveill.* 19:20858. 10.2807/1560-7917.es2014.19.29.20858 25080140

[B156] Oz F. Eksioglu A. Tanır G. Bayhan G. Metin O. Teke T. A. (2014). Evaluation of clinical and sonographic features in 55 children with tularemia. *Vector Borne Zoonotic Dis. Larchmt. N* 14 571–575. 10.1089/vbz.2013.1517 25072987

[B157] Ozkok A. Karadenizli A. Odabas A. R. (2012). Tularemia in a kidney transplant recipient. *Am. J. Kidney Dis.* 60:679. 10.1053/j.ajkd.2012.06.023 22901596

[B158] Pärssinen O. Rummukainen M. (1997). Acute glaucoma and acute corneal oedema in association with tularemia. *Acta Ophthalmol. Scand.* 75 732–734. 10.1111/j.1600-0420.1997.tb00642.x 9527343

[B159] Passiouk N. Heininger U. (2016). Ulceroglandular Tularemia following contact with a boar. *Pediatr. Infect. Dis. J.* 35 453–455. 10.1097/INF.0000000000001027 26658527

[B160] Pavlov V. M. Mokrievich A. N. Volkovoy K. (1996). Cryptic plasmid pFNL10 from *Francisella* novicida-like F6168: the base of plasmid vectors for *Francisella tularensis*. *FEMS Immunol. Med. Microbiol.* 13 253–256. 10.1111/j.1574-695X.1996.tb00247.x 8861039

[B161] Pekova L. M. Baymakova M. P. Parousheva P. O. Fartunova M. D. Dimitrov N. E. Tomova I. A. (2017). Clinical characteristics of Ulceroglandular Tularemia in two Bulgarian regions, 2014-2015: a report of five cases. *Folia Med.* 59 486–493. 10.1515/folmed-2017-0050 29341950

[B162] Pérez-Castrillón J. L. Bachiller-Luque P. Martín-Luquero M. Mena-Martín F. J. Herreros V. (2001). Tularemia epidemic in Northwestern Spain: clinical description and therapeutic response. *Clin. Infect. Dis.* 33 573–576. 10.1086/322601 11462198

[B163] Petersen J. M. Carlson J. Yockey B. Pillai S. Kuske C. Garbalena G. (2009). Direct isolation of *Francisella* spp. from environmental samples. *Lett. Appl. Microbiol.* 48 663–667. 10.1111/j.1472-765X.2009.02589.x 19413814

[B164] Peterson J. W. Moen S. T. Healy D. Pawlik J. E. Taormina J. Hardcastle J. (2010). Protection afforded by Fluoroquinolones in animal models of respiratory infections with *Bacillus anthracis*, *Yersinia pestis*, and *Francisella tularensis*. *Open Microbiol. J.* 4 34–46. 10.2174/1874285801004010034 21127743 PMC2995158

[B165] Piercy T. Steward J. Lever M. S. Brooks T. J. G. (2005). In vivo efficacy of fluoroquinolones against systemic tularaemia infection in mice. *J. Antimicrob. Chemother.* 56 1069–1073. 10.1093/jac/dki359 16223941

[B166] Polat M. Karapınar T. Sırmatel F. (2018). Dermatological aspects of tularaemia: a study of 168 cases. *Clin. Exp. Dermatol.* 43 770–774. 10.1111/ced.13548 29761532

[B167] Ponderand L. Guimard T. Lazaro E. Dupuy H. Peuchant O. Roch N. (2023). Case studies and literature review of *Francisella tularensis*-related prosthetic joint infection. *Emerg. Infect. Dis.* 29 1118–1126. 10.3201/eid2906.221395 37209668 PMC10202857

[B168] Rawal H. Patel A. Moran M. (2017). Unusual case of prosthetic joint infection caused by *Francisella tularensis*. *BMJ Case Rep.* 2017:bcr2017221258. 10.1136/bcr-2017-221258 29025776 PMC5652388

[B169] Reintjes R. Dedushaj I. Gjini A. Jorgensen T. R. Cotter B. Lieftucht A. (2002). Tularemia outbreak investigation in Kosovo: case control and environmental studies. *Emerg. Infect. Dis.* 8 69–73. 11749751 10.3201/eid0801.010131 PMC2730257

[B170] Rimawi R. H. Shah K. B. Chowdhary R. A. Cook P. P. (2014). Hunting for tularaemia - a review of cases in North Carolina. *Zoonoses Public Health*. 62 159–164. 10.1111/zph.12114 24655540

[B171] Rotem S. Bar-Haim E. Cohen H. Elia U. Ber R. Shafferman A. (2012). Consequences of delayed ciprofloxacin and doxycycline treatment regimens against *Francisella tularensis* airway infection. *Antimicrob. Agents Chemother.* 56 5406–5408. 10.1128/AAC.01104-12 22850512 PMC3457351

[B172] Russell P. Eley S. M. Fulop M. J. Bell D. L. Titball R. W. (1998). The efficacy of ciprofloxacin and doxycycline against experimental tularaemia. *J. Antimicrob. Chemother.* 41 461–465. 9598777 10.1093/jac/41.4.461

[B173] Salit I. E. Liles W. C. Smith C. (2013). Tularemia endocarditis from domestic pet exposure. *Am. J. Med.* 126:e1. 10.1016/j.amjmed.2013.04.011 23910519

[B174] Saranovic M. Milic M. Radic I. Katanic N. Vujacic M. Gasic M. (2023). Tularemia in pregnant woman, Serbia, 2018. *Emerg. Infect. Dis.* 29 806–808. 10.3201/eid2904.221318 36958014 PMC10045689

[B175] Sarria J. C. Vidal A. M. Kimbrough R. C. Figueroa J. E. (2003). Fatal infection caused by *Francisella tularensis* in a neutropenic bone marrow transplant recipient. *Ann. Hematol.* 82 41–43. 10.1007/s00277-002-0570-4 12574964

[B176] Scheel O. Hoel T. Sandvik T. Berdal B. P. (1993). Susceptibility pattern of Scandinavian *Francisella tularensis* isolates with regard to oral and parenteral antimicrobial agents. *APMIS* 101 33–36. 8384458

[B177] Sencan I. Sahin I. Kaya D. Oksuz S. Ozdemir D. Karabay O. (2009). An outbreak of oropharyngeal tularemia with cervical adenopathy predominantly in the left side. *Yonsei Med. J.* 50 50–54. 10.3349/ymj.2009.50.1.50 19259348 PMC2649863

[B178] Şenel E. Satılmış Ö Acar B. (2015). Dermatologic manifestations of tularemia: a study of 151 cases in the mid-Anatolian region of Turkey. *Int. J. Dermatol.* 54 e33–e37. 10.1111/ijd.12431 25208535

[B179] Sheshko V. Link M. Golovliov I. Balonova L. Stulik J. (2021). Utilization of a tetracycline-inducible system for high-level expression of recombinant proteins in *Francisella tularensis* LVS. *Plasmid* 115:102564. 10.1016/j.plasmid.2021.102564 33610608

[B180] Siebert C. Lindgren H. Ferré S. Villers C. Boisset S. Perard J. (2019). *Francisella tularensis*: FupA mutation contributes to fluoroquinolone resistance by increasing vesicle secretion and biofilm formation. *Emerg. Microbes Infect.* 8 808–822. 10.1080/22221751.2019.1615848 31164053 PMC6566608

[B181] Sjöstedt A. (2007). Tularemia: history, epidemiology, pathogen physiology, and clinical manifestations. *Ann. N. Y. Acad. Sci.* 1105 1–29. 10.1196/annals.1409.009 17395726

[B182] Sobolewska-Pilarczyk M. Pawłowska M. Halota W. (2014). Ulceroglandular tularemia complicated by pneumonia–a case report. *Przegl. Epidemiol.* 68 531–534. 25391005

[B183] Steinemann T. L. Sheikholeslami M. R. Brown H. H. Bradsher R. W. (1999). Oculoglandular tularemia. *Arch. Ophthalmol.* 117 132–133. 9930180 10.1001/archopht.117.1.132

[B184] Steward J. Piercy T. Lever M. S. Simpson A. J. H. Brooks T. J. G. (2006). Treatment of murine pneumonic *Francisella tularensis* infection with gatifloxacin, moxifloxacin or ciprofloxacin. *Int. J. Antimicrob. Agents* 27 439–443. 16621457 10.1016/j.ijantimicag.2006.02.006

[B185] Stundick M. V. Albrecht M. T. Houchens C. R. Smith A. P. Dreier T. M. Larsen J. C. (2013). Animal models for *Francisella tularensis* and *Burkholderia* species: scientific and regulatory gaps toward approval of antibiotics under the FDA animal rule. *Vet. Pathol.* 50 877–892. 10.1177/0300985813486812 23628693

[B186] Sutera V. Caspar Y. Boisset S. Maurin M. (2014a). A new dye uptake assay to test the activity of antibiotics against intracellular *Francisella tularensis*. *Front. Cell. Infect. Microbiol.* 4:36. 10.3389/fcimb.2014.00036 24672776 PMC3957058

[B187] Sutera V. Levert M. Burmeister W. P. Schneider D. Maurin M. (2014b). Evolution toward high-level fluoroquinolone resistance in *Francisella* species. *J. Antimicrob. Chemother.* 69 101–110. 10.1093/jac/dkt321 23963236

[B188] Sutera V. Hennebique A. Lopez F. Fernandez N. Schneider D. Maurin M. (2020). Genomic trajectories to fluoroquinolone resistance in *Francisella tularensis* subsp. *holarctica* live vaccine strain. *Int. J. Antimicrob. Agents* 56:106153. 10.1016/j.ijantimicag.2020.106153 32911069

[B189] Svensson K. Back E. Eliasson H. Berglund L. Granberg M. Karlsson L. (2009). Landscape epidemiology of Tularemia outbreaks in Sweden. *Emerg. Infect. Dis.* 15 1937–1947. 10.3201/eid1512.090487 19961673 PMC3044527

[B190] Syrjälä H. Karvonen J. Salminen A. (1984). Skin manifestations of tularemia: a study of 88 cases in northern Finland during 16 years (1967-1983). *Acta Derm. Venereol.* 64 513–516. 6084923

[B191] Tancik C. A. Dillaha J. A. (2000). *Francisella tularensis* endocarditis. *Clin. Infect. Dis.* 30 399–400. 10671352 10.1086/313678

[B192] Tärnvik A. Chu M. C. (2007). New approaches to diagnosis and therapy of tularemia. *Ann. N. Y. Acad. Sci.* 1105 378–404. 17468229 10.1196/annals.1409.017

[B193] Telford S. R. Goethert H. K. (2020). Ecology of *Francisella tularensis*. *Annu. Rev. Entomol.* 65 351–372. 31600457 10.1146/annurev-ento-011019-025134 PMC8300880

[B194] Terrada C. Azza S. Bodaghi B. Le Hoang P. Drancourt M. (2016). Rabbit hunter uveitis: case report of tularemia uveitis. *BMC Ophthalmol.* 16:157. 10.1186/s12886-016-0332-z 27585457 PMC5009509

[B195] Tezer H. Ozkaya-Parlakay A. Aykan H. Erkocoglu M. Gülhan B. Demir A. (2015). Tularemia in children, Turkey, September 2009-November 2012. *Emerg. Infect. Dis.* 21 1–7. 10.3201/eid2101.131127 25529639 PMC4285238

[B196] Tezer M. S. Övet G. Alataş N. Görgülü M. H. Koç E. Öztürk M. A. (2013). Clinical manifestations of 16 oropharyngeal tularemia patients: experience of a referral hospital in the city of Konya, Turkey. *Turk. J. Med. Sci.* 43 227–231.

[B197] Thomas L. D. Schaffner W. (2010). Tularemia pneumonia. *Infect. Dis. Clin. North Am.* 24 43–55. 20171544 10.1016/j.idc.2009.10.012

[B198] Timofeev V. Bakhteeva I. Titareva G. Kopylov P. Christiany D. Mokrievich A. (2017). Russian isolates enlarge the known geographic diversity of *Francisella tularensis* subsp. *mediasiatica*. *PLoS One* 12:e0183714. 10.1371/journal.pone.0183714 28873421 PMC5584958

[B199] Tomaso H. Al Dahouk S. Hofer E. Splettstoesser W. D. Treu T. M. Dierich M. P. (2005). Antimicrobial susceptibilities of Austrian *Francisella tularensis holarctica* biovar II strains. *Int. J. Antimicrob. Agents* 26 279–284. 10.1016/j.ijantimicag.2005.07.003 16143497

[B200] Tomaso H. Hotzel H. Otto P. Myrtennäs K. Forsman M. (2017). Antibiotic susceptibility in vitro of *Francisella tularensis* subsp. *holarctica* isolates from Germany. *J. Antimicrob. Chemother.* 72 2539–2543. 10.1093/jac/dkx182 28605439

[B201] Ughetto E. Héry-Arnaud G. Cariou M.-E. Pelloux I. Maurin M. Caillon J. (2015). An original case of *Francisella tularensis* subsp. *holarctica* bacteremia after a near-drowning accident. *Infect. Dis. Lond. Engl.* 47 588–590. 10.3109/23744235.2015.1028099 25816922

[B202] Ulu-Kilic A. Gulen G. Sezen F. Kilic S. Sencan I. (2013). Tularemia in central anatolia. *Infection* 41 391–399. 23104256 10.1007/s15010-012-0355-1

[B203] Urich S. K. Petersen J. M. (2008). In vitro susceptibility of isolates of *Francisella tularensis* types A and B from North America. *Antimicrob. Agents Chemother.* 52 2276–2278. 10.1128/AAC.01584-07 18411318 PMC2415761

[B204] Uzun M. Ö Yanik K. Erdem M. Kostakoglu U. Yilmaz G. Tanriverdi Çayci Y. (2015). Epidemiological and clinical characteristics and management of oropharyngeal tularemia outbreak. *Turk. J. Med. Sci.* 45 902–906. 10.3906/sag-1403-111 26422865

[B205] Väyrynen S. A. Saarela E. Henry J. Lahti S. Harju T. Kauma H. (2017). Pneumonic tularaemia: experience of 58 cases from 2000 to 2012 in Northern Finland. *Infect. Dis. Lond. Engl.* 49 758–764. 10.1080/23744235.2017.1341054 28618894

[B206] Velinov T. Kantardjiev T. Nicolova Kuzmanov. (2011). In vitro antimicrobial susceptibility of *Francisella tularensis* isolated in Bulgaria. *Probl. Infect. Parasit. Dis.* 39 7–9.

[B207] Venkatesan S. Johnston C. Mehrizi M. Z. (2020). A rare case of tularemic meningitis in the United States from aerosolized *Francisella tularensis*. *J. Am. Coll. Emerg. Physicians Open* 1 238–241. 10.1002/emp2.12037 33000038 PMC7493567

[B208] Vogler A. J. Birdsell D. Wagner D. M. Keim P. (2009). An optimized, multiplexed multi-locus variable-number tandem repeat analysis system for genotyping *Francisella tularensis*. *Lett. Appl. Microbiol.* 48 140–144. 10.1111/j.1472-765X.2008.02484.x 19018964

[B209] Wang X. Ribeiro A. A. Guan Z. Abraham S. N. Raetz C. R. H. (2007). Attenuated virulence of a *Francisella* mutant lacking the lipid A 4’-phosphatase. *Proc. Natl. Acad. Sci. U. S. A.* 104 4136–4141. 10.1073/pnas.0611606104 17360489 PMC1820721

[B210] Wawszczak M. Banaszczak B. Rastawicki W. (2022). Tularaemia - a diagnostic challenge. *Ann. Agric. Environ. Med. AAEM* 29 12–21. 35352900 10.26444/aaem/139242

[B211] Weber I. B. Turabelidze G. Patrick S. Griffith K. S. Kugeler K. J. Mead P. S. (2012). Clinical recognition and management of tularemia in Missouri: a retrospective records review of 121 cases. *Clin. Infect. Dis.* 55 1283–1290. 10.1093/cid/cis706 22911645

[B212] Weile J. Seibold E. Knabbe C. Kaufmann M. Splettstoesser W. (2013). Treatment of tularemia in patient with chronic graft-versus-host disease. *Emerg. Infect. Dis.* 19 771–773. 10.3201/eid1905.120377 23647853 PMC3647492

[B213] Weiner E. Stryjewski G. Eppes S. (2004). Variable presentation of the cause of lymphadenopathy in two children. *Pediatr. Infect. Dis. J.* 23 972–973. 15602207 10.1097/01.inf.0000142010.24123.7d

[B214] Wetzstein N. Kärcher I. Küpper-Tetzel C. P. Kann G. Hogardt M. Jozsa K. (2019). Clinical characteristics in a sentinel case as well as in a cluster of tularemia patients associated with grape harvest. *Int. J. Infect. Dis.* 84 116–120. 10.1016/j.ijid.2019.04.031 31071480

[B215] Whitsell N. W. Becker H. (2020). Tularemia hand infection from a cat bite-A case report. *J. Hand Surg. Glob. Online* 2 320–322. 10.1016/j.jhsg.2020.07.001 35415518 PMC8991864

[B216] Whitten T. Bjork J. Neitzel D. Smith K. Sullivan M. Scheftel J. (2017). Notes from the field: *Francisella tularensis* Type B infection from a fish hook injury - Minnesota, 2016. *MMWR* 66:194. 10.15585/mmwr.mm6607a3 28231234 PMC5657846

[B217] Willke A. Meric M. Grunow R. Sayan M. Finke E. J. Splettstösser W. (2009). An outbreak of oropharyngeal tularaemia linked to natural spring water. *J. Med. Microbiol.* 58 112–116. 10.1099/jmm.0.002279-0 19074661

[B218] World Health Organization (1970). *Health Aspects of Chemical and Biological Weapons: Report of a WHO Group of Consultants.* Geneva: World Health Organization.

[B219] Yeom J. S. Rhie K. Park J. S. Seo J.-H. Park E. S. Lim J.-Y. (2015). The first pediatric case of tularemia in Korea: manifested with pneumonia and possible infective endocarditis. *Korean J. Pediatr.* 58 398–401. 10.3345/kjp.2015.58.10.398 26576185 PMC4644769

[B220] Yesilyurt M. Kiliç S. Celebi B. Celik M. Gül S. Erdogan F. (2011). Antimicrobial susceptibilities of *Francisella tularensis* subsp. *holarctica* strains isolated from humans in the Central Anatolia region of Turkey. *J. Antimicrob. Chemother.* 66 2588–2592. 10.1093/jac/dkr338 21856791

[B221] Yeşilyurt M. Kiliç S. Çelebi B. Gül S. (2013). Tularemia during pregnancy: report of four cases. *Scand. J. Infect. Dis.* 45 324–328. 10.3109/00365548.2012.720027 22998506

[B222] Zaw M. T. Emran N. A. Lin Z. (2018). Mutations inside rifampicin-resistance determining region of rpoB gene associated with rifampicin-resistance in *Mycobacterium tuberculosis*. *J. Infect. Public Health* 11 605–610. 10.1016/j.jiph.2018.04.005 29706316

